# Exploring island syndromes: Variable matrix permeability in *Phalaenopsis pulcherrima* (Orchidaceae), a specialist lithophyte of tropical Asian inselbergs

**DOI:** 10.3389/fpls.2023.1097113

**Published:** 2023-02-20

**Authors:** Zhe Zhang, Jihong Li, Somran Suddee, Somsanith Bouamanivong, Leonid V. Averyanov, Stephan W. Gale

**Affiliations:** ^1^ Key Laboratory of Genetics and Germplasm Innovation of Tropical Special Forest Trees and Ornamental Plants (Hainan University), Ministry of Education, College of Forestry, Hainan University, Haikou, China; ^2^ Key Laboratory of Germplasm Resources of Tropical Special Ornamental Plants of Hainan Province, College of Forestry, Hainan University, Haikou, China; ^3^ Flora Conservation Department, Kadoorie Farm & Botanic Garden, Tai Po, Hong Kong, China; ^4^ Forest Herbarium, Department of National Parks, Wildlife and Plant Conservation, Chatuchak, Bangkok, Thailand; ^5^ Biotechnology and Ecology Institute, Ministry of Science and Technology, Vientiane, Laos; ^6^ Komarov Botanical Institute, Russian Academy of Sciences, St. Petersburg, Russia

**Keywords:** gene flow, genetic structuring, historical demography, island syndrome, lithophyte, matrix permeability, terrestrial habitat islands, tropical Asian inselbergs

## Abstract

**Introduction:**

Plants confined to island-like habitats are hypothesised to possess a suite of functional traits that promote on-spot persistence and recruitment, but this may come at the cost of broad-based colonising potential. Ecological functions that define this island syndrome are expected to generate a characteristic genetic signature. Here we examine genetic structuring in the orchid *Phalaenopsis pulcherrima*, a specialist lithophyte of tropical Asian inselbergs, both at the scale of individual outcrops and across much of its range in Indochina and on Hainan Island, to infer patterns of gene flow in the context of an exploration of island syndrome traits.

**Methods:**

We sampled 323 individuals occurring in 20 populations on 15 widely scattered inselbergs, and quantified genetic diversity, isolation-by-distance and genetic structuring using 14 microsatellite markers. To incorporate a temporal dimension, we inferred historical demography and estimated direction of gene flow using Bayesian approaches.

**Results:**

We uncovered high genotypic diversity, high heterozygosity and low rates of inbreeding, as well as strong evidence for the occurrence of two genetic clusters, one comprising the populations of Hainan Island and the other those of mainland Indochina. Connectivity was greater within, rather than between the two clusters, with the former unequivocally supported as ancestral.

**Discussion:**

Despite a strong capacity for on-spot persistence conferred by clonality, incomplete self-sterility and an ability to utilize multiple magnet species for pollination, our data reveal that *P. pulcherrima* also possesses traits that promote landscape-scale gene flow, including deceptive pollination and wind-borne seed dispersal, generating an ecological profile that neither fully conforms to, nor fully contradicts, a putative island syndrome. A terrestrial matrix is shown to be significantly more permeable than open water, with the direction of historic gene flow indicating that island populations can serve as refugia for postglacial colonisation of continental landmasses by effective dispersers.

## 1 Introduction

Explanations for the assembly and composition of island biotas have been widely applied to biogeographic patterns observed across a range of island-like systems (Liira et al., 2014; Adams et al., 2017; Henneron et al., 2019). However, models that have emerged as fundamental to characterising the ecology and evolution of life on true islands – including species–isolation relationships, community nestedness and genetic divergence (MacArthur and Wilson, 1967; Carlquist, 1974; Burns, 2019) – have proven to be of variable utility in understanding biological processes in other habitats that conform to a biological definition of insularity (Itescu, 2019). Thus, whilst habitat patches that are separated from one another by some form of geographic or physiological barrier tend to experience similar eco-evolutionary constraints that demarcate them from their surrounding matrix, connectivity is often greater than that over open water, especially in the case of terrestrial habitat islands (Itescu, 2019; Ottaviani et al., 2020). Not only has this sparked debate as to whether terrestrial island-like systems really function as true islands (Mendez-Castro et al., 2021), it has also fomented effort to systematise classifications of insularity and focused attention on the ecological functions and underlying traits that govern performance in habitat fragments (Itescu, 2019; Ottaviani et al., 2020). Ottaviani et al. (2020) hypothesise that suites of functional traits manifest in plants that inhabit insular systems – that is, island syndromes – will differ between true islands and terrestrial habitat islands. They advocate research to explore this hypothesis.

Traits central to defining a hypothetical island syndrome relate primarily to the ability of individuals to persist in confined environments (Ottaviani et al., 2020). This is because the abiotic and biotic conditions that prevail in insular systems are expected to impose an array of ecological filters which, acting together with geographic isolation, select for life history attributes that promote on-spot persistence and recruitment (Fine and Baraloto, 2016). With increasing isolation, plants specialised to islands and island-like habitats are predicted to exhibit weaker dispersal potential, higher clonality and greater reliance on selfing, as compared with those occurring in mainland or more widespread matrix environments. Several studies have shown this to be the case (e.g., Lhuillier et al., 2006; Grossenbacher et al., 2017). However, the prominence of this effect across a variety of terrestrial island-like systems, among which the permeability of the surrounding matrix differs for different species or functional groups, remains to be examined.

Many of the ecological functions that define island trait syndromes are expected to generate characteristic signatures in terms of genetic structuring and gene flow (Rossetto et al., 2008; Gao et al., 2015). Thus, the balance of vegetative growth versus sexual recruitment, and of selfing as opposed to outcrossing, will impact genetic diversity indices, and pollination and seed dispersal strategies will influence average gene flow distances and divergence (Auffret et al., 2017). Indeed, studies of plants restricted to terrestrial island habitats have uncovered trade-offs between on-site persistence and genetic diversity (Hmeljevski et al., 2014; Lozada-Gobilard et al., 2021), pollinator sharing and genetic differentiation (Wanderley et al., 2018), pollen flow and genetic structuring (Lexer et al., 2016), and seed dispersal and genetic divergence (Rossetto et al., 2008; Gao et al., 2015). In some cases, these findings have been taken as evidence of the applicability of island biogeography theories to terrestrial habitat islands, with species that are more geographically isolated and more confined by habitat-specificity typically being found to exhibit an array of traits consistent with an island syndrome (e.g., fibrous fruits, gravity-dispersed seeds, strong resprouting behaviour, self-compatibility and pollinator specialisation). They also suggest that specialisation to a terrestrial island-like system could come at the cost of broad-based colonising potential.

Even so, more research is needed before an understanding of the significance of different ecological strategies can be synthesised from a representative cross-section of insular systems (Ottaviani et al., 2020). One system that remains vastly under-studied in this respect is tropical Asia’s network of granitic and gneissic inselbergs. Unlike similar outcrops elsewhere (Porembski and Barthlott, 2000), no ecological trait-based or genetic studies of plants confined to this island-like system have previously been undertaken. As such, information on the extent to which they exhibit typical island-like features is lacking. Given the disturbance and destruction to which they are increasingly subjected due to quarrying, agriculture and plantation forestry, studies relevant to the conservation of their fragile floras are urgently needed.

In describing the flora of a granitic and sandstone dome in Peninsular Thailand, Inuthai and Sridith (2010) present the only published account of the vegetation of a tropical Asian inselberg. They recognise seven microhabitats based on discrete topographic features and identify all vascular plants occurring in each. Their classification largely coincides with habitat types recognised on inselbergs in other regions (Kluge and Büdel, 2009) and underscores the observation that specific microhabitats accommodate unique communities of plants with particular adaptive traits (Porembski and Barthlott, 2000). The most species-rich family was Orchidaceae, mirroring a trend reported for inselbergs elsewhere (Porembski and Barthlott, 2000; Gomes and Alves, 2009) and suggesting that certain orchid lineages are well suited to life in the xeric conditions that prevail on inselbergs, owing to their root velamen, succulent leaves and CAM photosynthesis (Chomicki et al., 2015; Zhang et al., 2018). The lithophytic orchid *Phalaenopsis pulcherrima* (Lindl.) J.J.Sm. was consistently associated with shallow depressions and rock platform fringes (Inuthai and Sridith, 2010).

In contrast to all other members of *Phalaenopsis*, a genus of ca. 75 epiphytic species occurring in tropical Asia, New Guinea and Australia (Govaerts et al., 2021), *P. pulcherrima* forms dense clonal clumps, has prop-like, adventitious roots that anchor its upright stem directly to rocky substrates, it bears clustered, succulent leaves and produces erect inflorescences. Despite being native to most countries of Southeast Asia (Christenson, 2001), the species has a highly scattered occurrence, reflecting a specific requirement for open granitic shelves or, rarely, coarse, weathered quartzite soils (Averyanov, 2009; Zhang et al., 2019). As a characteristic element of tropical Asian inselbergs, *P. pulcherrima* constitutes an excellent model for examining colonisation of, and persistence within, this terrestrial island-like system. Here, we elucidate population genetic structure, both at the scale of individual inselbergs and across much of its range, to infer patterns of gene flow and the degree to which the surrounding matrix constrains dispersal, in the context of an exploration of island syndrome traits. Given its strict association with scattered granitic platforms, we hypothesise that the species will exhibit a genetic signature typical of terrestrial habitat island plants and thus help refine understanding of an island syndrome. Specifically, we ask: (i) how genetically diverse is *P. pulcherrima* at a range of spatial scales, (ii) how ecologically inter-connected are its geographically isolated populations, and (iii) to what extent do underlying functional traits determining its dispersal, persistence and reproductive strategy conform to a putative island syndrome?

## 2 Materials and methods

### 2.1 Study species

**Figure 1:**
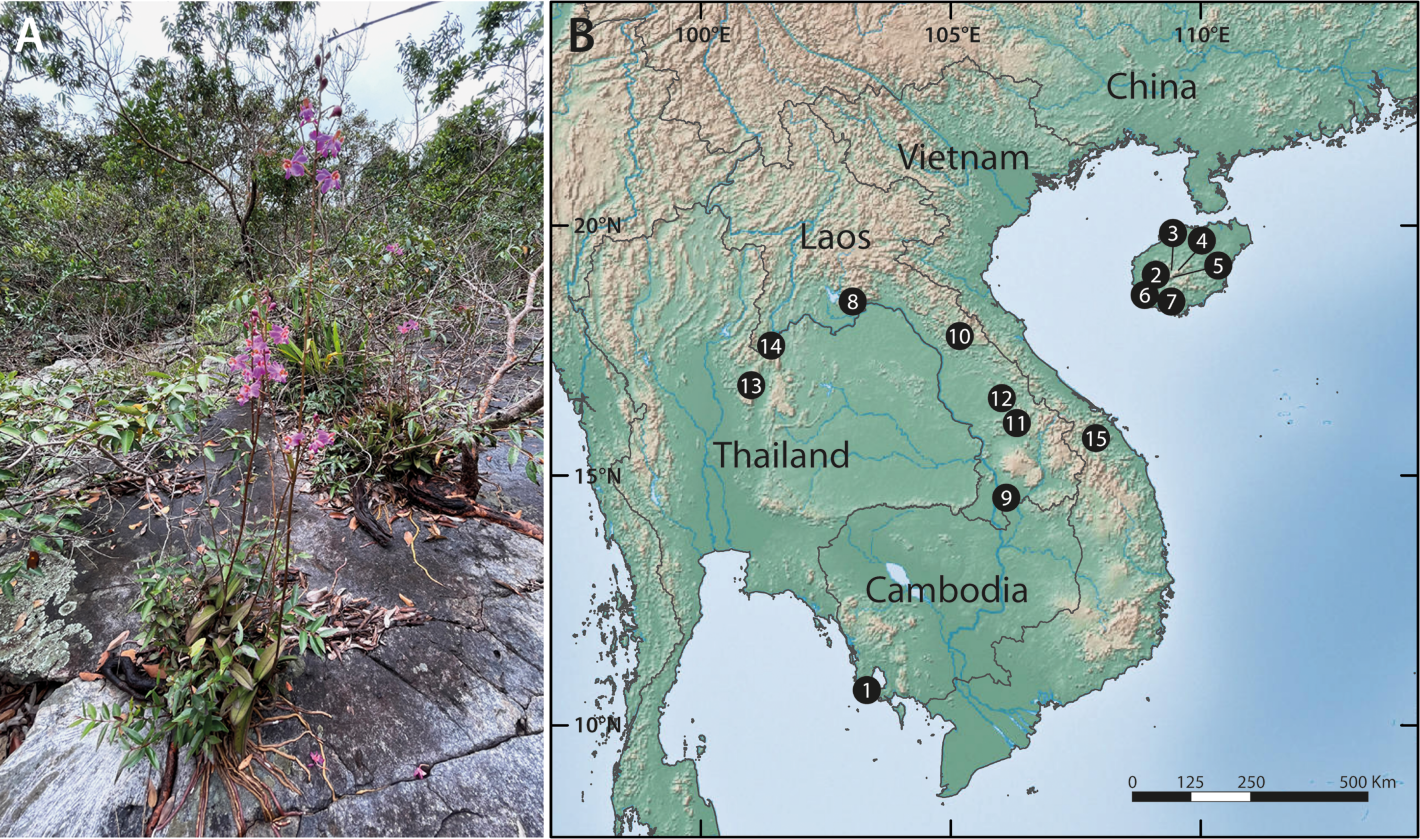
Typical habit and habitat of *Phalaenopsis pulcherrima* and geographic scope of the study. **(A)** Plants growing in a shallow depression on a granitic inselberg in northeast Thailand. Note the long, velamentous roots directly attached to the substrate. Photo: T. Sando. **(B)** Location of the 15 inselbergs included in this study; see **Table 1** for information on the populations sampled at each site.

*Phalaenopsis pulcherrima* is a lithophytic herb that is distributed primarily in the seasonal Asian tropics, from northeast India and Myanmar, through Thailand to Indochina and Hainan Island in South China, with a few outlying localities in Peninsular Malaysia, Sumatra and Borneo (Christenson, 2001; Kumar et al., 2018). Despite this wide range, the species is rare, being confined to open granitic platforms in monsoon forest (**Figure 1A**). Jin et al. (2012) found it to achieve pollination through generalised food-deception of solitary bees and Zhang et al. (2019) demonstrated that this, plus self-sterility, promote outcrossing and landscape-scale pollen flow, thereby enabling clusters occurring on the same inselberg to remain genetically connected. Although this generated high diversity at the population level, fine-scale structure was detected as a result of clonal propagation and localised gene flow *via* seed (Zhang et al., 2019). More recently, Hu et al. (2021) suggested that green- and red-coloured leaf morphs might be genetically differentiated, but sampling among morphs and across populations was low and inconsistent. The species’ ecological confinement to terrestrial habitat islands, as well as its geographic occurrence on both mainland and true island landmasses, makes it an ideal subject for investigating landscape permeability and colonisation history.

### 2.2 Sampling, DNA extraction and SSR genotyping

We mapped and sampled all 323 individuals occurring in 20 populations on 15 inselbergs scattered across a large swathe of the species’ native range in Thailand, Laos, Cambodia, Vietnam and South China (**Figure 1B** and **Table 1**). Each population contained between two and 60 individuals. Physically connected shoots and plants occurring within 10 cm of one another were avoided to minimise sampling ramets belonging to the same clone. All field work and plant material collection complied with relevant institutional, national and international restrictions (CITES permits: 004/16-01, 005/16, 006/16-01, 2016 TH 001913/BE). Fresh leaf material was immediately placed in silica gel and stored at -80°C once desiccated. Total genomic DNA was extracted from the dried samples using a modified CTAB protocol. All samples were then screened with 14 microsatellite markers developed by, and in accordance with, the methodology of Zhang et al. (2019). PCR products were resolved on an ABI3730xl Genetic Analyzer (Applied Biosystems, Foster City, USA) with an internal LIZ (500) size standard and fragment data were analysed using GENEMARKER ver. 2.4.0 (Softgenetics LLC, State College, USA).

**Table 1:** Populations included in this study and number of individuals sampled at each.

Country	Population (inselberg location in Figure 1B)	Population code	No. of plants sampled	Voucher specimen
Cambodia	Sihanoukville Province, Koh Rong Sanloem (1)	KRS	2	*Maisak et al. 1018* (LE)
China	Hainan Province, Bawangling, Wangxia – 1 (2)	WX1	56	*Zhang & Song PP2011080811* (HUTB)
	Hainan Province, Bawangling, Wangxia – 2 (2)	WX2	39	*Zhang & Song PP2011080812* (HUTB)
	Hainan Province, Bawangling, Wangxia – 3 (2)	WX3	60	*Zhang & Song PP2011080813* (HUTB)
	Hainan Province, Bawangling, Yajia (3)	YJ	12	*Si & Song PP2010070512* (HUTB)
	Hainan Province, Bawangling, Dongliu (4)	DL	5	*Si & Song PP2010070601* (HUTB)
	Hainan Province, Bawangling, Dongliu hydroelectric plant (5)	DLH	5	*Si & Song PP2010070705* (HUTB)
	Hainan Province, Jianfengling (6)	JF	6	*Si & Song PP2010071222* (HUTB)
	Hainan Province, Ledong – 1 (7)	LD1	16	*Si & Song PP2010072502* (HUTB)
	Hainan Province, Ledong – 2 (7)	LD2	9	*Si & Song PP2010072501* (HUTB)
Laos	Bolikhamxay Province, Thaphabath District, Phou Khao Khouay (8)	PKK	13	*Gale et al. PKK2016A* (KFBG)
	Champasak Province, Phatoumphone District, Xe Pian (9)	XP	4	*Gale et al. HNL-KFBG1104* (HNL)
	Khammouane Province, Nakai-Nam Theun Protected Area – 1 (10)	NPA1	4	*Gale et al. HNL-KFBG319* (HNL)
	Khammouane Province, Nakai-Nam Theun Protected Area – 2 (10)	NPA2	40	*Gale et al. HNL-KFBG321* (HNL)
	Savannakhet Province, Phin District, Dong Phou Vieng (11)	DPV	2	*Gale et al. HNL-KFBG888* (HNL)
	Savannakhet Province, Phou Xiang He – 1 (12)	PXH1	23	*Gale et al. PXH2016B* (KFBG)
	Savannakhet Province, Phou Xiang He – 2 (12)	PXH2	7	*Gale et al. PXH2016C* (KFBG)
Thailand	Phitsanulok Province, Phu Hin Rong Kla (13)	PHR	9	*Suddee 5050* (BKF)
	Loei Province, Phu Ruea (14)	PR	9	*Suddee 5054* (BKF)
Vietnam	Dac Lac Province, Buon Don District, Yok Don National Park (15)	YD	2	*Hiep et al. HLF7340* (LE)
		Total	323	

The number in parentheses after the population name indicates the inselberg location as shown in **Figure 1B**.

### 2.3 Genetic diversity and differentiation

We checked for the presence of null alleles using MICRO-CHECKER ver. 2.2.3 (van Oosterhout et al., 2004) and departure from Hardy-Weinberg equilibrium using GENEPOP ver. 4.2, with all Markov chain parameters (dememorization, number of batches and number of iterations per batch) set to 10,000 (Raymond and Rousset, 1995). We then calculated the following summary statistics for the 14 loci using GenAlEx ver. 6.5 (Peakall and Smouse, 2012): total number of alleles (*Nt*), number of alleles per locus (*Na*), number of effective alleles (*Ne*), total heterozygosity (*Ht*), observed heterozygosity (*Ho*), expected heterozygosity (*He*), Shannon’s information index (*I*), Wright’s F statistics (*Fis*, *Fit* and *Fst*), number of private alleles (*Np*), gene flow (*Nm*) and proportion of polymorphic loci (*PPL*). Additionally, standardised *Fst* (*F′st* = *Fst/Fstmax*) (Hedrick, 2005) was calculated from *Fst* and *Fst* maximum values generated using FSTAT version 2.9.3 (Goudet, 1995) and RECODEDATA for data recoding (Meirmans, 2006).

### 2.4 Genetic structure and cluster analysis

A neighbour-joining (NJ) tree was calculated in POPTREEW (Takezaki et al., 2014) and constructed in MEGA ver. X (Tamura et al., 2013) from a matrix of Nei’s genetic distances between populations. To better assess genetic relationships, we conducted principal coordinate analysis (PCoA) of mean pairwise genetic distances between populations in GenAlEx 6.5. We also determined the level of genetic differentiation among populations using *Fst* and analysis of molecular variance (AMOVA) with 1,000 permutations in GenAlEx 6.5.

Population genetic structure was evaluated across all alleles using STRUCTURE 2.3.3 (Pritchard et al., 2000), with prior information on assignment to population clusters derived from an extended Bayesian analysis (LOCPRIOR model). Delta K was developed and tested to prove the true population structure under different simulation routines. Delta K indicated a clear peak at the true value of K. An admixture model was used with ten iterations per K value ranging from 1 to 10 assuming correlated allele frequencies, with 1,000,000 Markov chain Monte Carlo (MCMC) repetitions after a burn-in period of 100,000 iterations. We adopted the height of Delta K as an indicator of the strength of the signal, as determined in STRUCTURE HARVESTER (Evanno et al., 2005; Earl and vonHoldt, 2012), and we used Clustering Markov Packager Across K (CLUMPAK) to visualise the results. A Mantel test was performed in GenAlEx 6.5 between Nei’s genetic distance and geographic distance (km) to quantify isolation-by-distance (IBD) among populations. Significance was evaluated with 1,000 randomisations.

### 2.5 Gene flow and population demography analyses

Based on the results of the STRUCTURE, PCoA and NJ analyses, the 20 populations were divided into a Hainan Island group (HN, containing nine populations) and a mainland Indochina group (IC, containing 11 populations), within which we checked for evidence of genetic bottlenecks at both the population and group level. This was done using BOTTLENECK ver. 1.3.2 (Piry et al., 1999). Of the three candidate mutation models, we applied the two-phased mutation model (TPM) because it is most suitable for microsatellite data; 1,000 replications were run, and a Wilcoxon sign rank test was used to evaluate significance (Di Rienzo et al., 1994). Alleles were classified into ten frequency classes, with the resulting distribution being assessed against a normal L-shaped form.

To estimate the direction of gene flow, we assessed the data against six possible models in MIGRATE 4 (Beerli, 2009): (M1) IC populations are offspring of HN populations with gene flow from HN to IC; (M2) HN populations are offspring of IC populations with gene flow from IC to HN; (M3) HN populations are offspring of IC populations with no gene flow between them; (M4) IC populations are offspring of HN populations with no gene flow between them; (M5) HN populations are offspring of IC populations with free gene flow between them; and (M6) IC populations are offspring of HN populations with free gene flow between them. For each model, a marginal likelihood and Bayes factor were calculated to infer the direction of migration and corresponding probability, with the number of steps in the chain set to 1,000,000.

We next employed DIYABC ver. 2.0 to assess the historical demography and ancestral distribution of *P. pulcherrima*, based on an approximate Bayesian computation (ABC) algorithm (Cornuet et al., 2014). We set prior values for the effective population size and divergence time estimates to give a uniform distribution for all parameters, generating a reference table based on 3 × 10^6^ simulated datasets (**Supplementary File S1**). A uniform prior distribution and a generalised stepwise mutation model were assumed, with a mean mutation rate of 5 × 10^-4^, which ranged from 10^-4^ to 10^-3^ mutations per generation per locus, as per the 95% CI in the initial run. Using the 1% of simulated datasets that approximated closest to observed data, we estimated relative posterior probabilities (PP) for three possible divergence scenarios: (S1) IC group originated from HN group and diverged at t1; (S2) HN group originated from IC group and diverged at t1; and (S3) both HN and IC groups originated from a single combined HN+IC ancestral population and diverged at t1 (**Supplementary File S2A**). To examine the extent and timing of historical population contractions and expansions, we also applied a logistic regression approach to assign posterior probabilities to the following nine potential demographic scenarios: (DS1) expansion + contraction model, Ne < NA; (DS2) expansion + contraction model, Ne > Na; (DS3) contraction + expansion model, Ne > Na; (DS4) old contraction model, Ne < NA; (DS5) old expansion model, Ne > Na; (DS6) contraction + expansion model, Ne < NA; (DS7) old expansion + intermediate contraction + recent expansion, Ne < NA, Ne > Nc > Na; (DS8) old expansion + intermediate contraction + recent expansion, Ne < NA, Ne > Na > Nb; (DS9) old contraction + intermediate expansion + recent contraction, Ne > Na > Nb (Cornuet et al., 2010; **Supplementary File S2B**). Posterior estimates of historical demographic parameters were evaluated by determining best-fit among simulated and real datasets using principal component analysis (PCA; **Supplementary Files S2C, D**). Finally, we estimated the specific time values of t1 and t2 by incorporating generation time into the results of the ABC model. To do so, we inferred a generation length of 5 years, in line with our field observations (Zhang et al., 2019) and seed germination experiments (Gale et al., 2019).

### 2.6 Fine-scale genetic structure and dispersal estimates

We ran spatial auto-correlation analysis in SPAGEDI (Hardy and Vekemans, 2002) to test for the presence of fine-scale genetic structure at the five populations with more than 20 individuals (WX1, WX2, WX3, NPA2 and PXH1). Pairwise kinship coefficients were calculated between all individuals (*F_ij_*) within each population (Loiselle et al., 1995; Kalisz et al., 2001), mean *F_ij_* was derived for each distance interval, *d*, and this was plotted against distance in metres. The software requires that the number of pairwise comparisons is kept constant across all distance intervals. Mean *F_ij_* (*d*) estimates were calculated for intervals defined as 0–20 m (*d* = 5 m), 21–50 m (*d* = 10 m), 51–100 m (*d* = 50 m or end-point) and 101–600 m (*d* = 100 m or end-point), and 95% confidence intervals (CI) associated with the null hypothesis of no genetic structure [*F_ij_* (*d*) = 0] were constructed using 1,000 random permutations. Significant positive or negative structure was inferred if the CIs did not overlap.

We then regressed the slope *b*_LF(_ *_d_*_)_ [linear regression of *F_ij_* (*d*) on ln (*d*)] to test whether there was significant deviation from the null hypothesis of no genetic structure [*b*_LF(_ *_d_*_)_ = 0]. To compare overall intensity of fine-scale genetic structure among populations, we also calculated the *Sp* statistic (Vekemans and Hardy, 2004), given by *Sp* = -*b*_LF(_ *_d_*_)_/[1-F (*d_1_*)], where F(*d_1_*) is the average kinship coefficient between individuals of the first distance class (i.e., 0–20 m, *d* = 5 m), *F_ij_*.

Finally, we estimated the relative contribution of pollen (σ_p_) and seed (σ_s_) dispersal to total gene flow, σ (Heuertz, 2010). Using the average *F_ij_* (*d*) for all samples from each population, we regressed the residuals [*f*(*d*): *F_ij_* (*d*) - *F_ij_* (*d*)_exp_] on ln(*d*) by a polynomial regression of the third power: *f*(*d*) = a + b ln(*d*) + c [ln(*d*)]^2^ + d [ln(*d*)]^3^, where *F_ij_* (*d*)_exp_ is the dependent variable of the linear regression equation at independent variable ln(*d*). The curvature of *f*(*d*) is given by the second derivative, *k* = 2c + 6d*ln (*d*_1_), where *d*_1_ is the average distance of the first distance class. A concave curve at short distances or *k >*0 suggests more restricted seed dispersal than pollen dispersal (σ_s_ ≪ σ_p_), whereas a convex shape or *k <*0 suggests more restricted pollen dispersal or no particular restriction in seed dispersal (σ_s_ ≥ σ_p_) (Vekemans and Hardy, 2004). Statistics were calculated in SPAGEDI (Hardy and Vekemans, 2002) and SPSS 22.0 (IBM Corp., New York, USA).

## 3 Results

### 3.1 Genotypic diversity

No null alleles were detected at any of the 14 microsatellite loci and high genetic diversity was confirmed (**Table 2**). A total of 207 alleles were detected across all samples. *Nt* ranged from 4 (L53) to 27 (L33) with a mean of 14.786; *Na* ranged from 1.95 (L53) to 5.95 (L64) with a mean of 4.214; and *Ne* ranged from 1.612 (L53) to 4.131 (L33) with a mean of 2.918. Shannon’s Information Index ranged from 0.494 (L53) to 1.481 (L33) with a mean of 1.076; *Ho* and *He* ranged from 0.392 (L22) to 0.840 (L54) and from 0.328 (L53) to 0.732 (L33), with means of 0.644 and 0.568, respectively. *He* was lower than *Ho* at 12 loci, with the two exceptions being L22 and L46 (*Fis* = 0.054 and 0.030, respectively) (**Table 2**).

**Table 2:** Genetic diversity metrics among all samples (n = 323) and across all 14 microsatellite loci used in this study.

Locus	*Nt*	*Na*	*Ne*	*Ho*	*He*	*Ht*	*I*	*Fis*	*Fit*	*Fst*	*Nm*	*F’s*t
L3	19	5.650	3.890	0.739	0.714	0.883	1.440	-0.036 ***	0.163	0.191	1.056	0.396
L6	14	4.050	3.180	0.674	0.585	0.828	1.109	-0.152 ^***^	0.186	0.293	0.603	0.486
L9	16	3.650	2.607	0.624	0.548	0.832	0.985	-0.139 ^***^	0.249	0.341	0.484	0.552
L22	10	2.800	1.934	0.392	0.415	0.679	0.693	0.054 ^***^	0.422	0.389	0.393	0.521
L29	15	4.850	3.037	0.661	0.595	0.881	1.165	-0.112 ^***^	0.250	0.325	0.519	0.650
L33	27	5.800	4.131	0.792	0.732	0.911	1.481	-0.082 ^***^	0.131	0.197	1.019	0.541
L46	24	5.300	3.639	0.624	0.644	0.882	1.306	0.030 ^***^	0.292	0.270	0.676	0.601
L51	11	2.850	1.893	0.415	0.390	0.816	0.699	-0.064 ^***^	0.491	0.522	0.229	0.418
L52	12	3.850	2.712	0.711	0.593	0.785	1.078	-0.200 ^***^	0.095	0.246	0.768	0.334
L53	4	1.950	1.612	0.541	0.328	0.403	0.494	-0.650 ^***^	-0.340	0.188	1.080	0.230
L54	13	4.900	3.486	0.840	0.692	0.867	1.338	-0.214 ^***^	0.032	0.203	0.983	0.502
L56	9	2.900	2.134	0.552	0.461	0.651	0.780	-0.199 ^***^	0.151	0.292	0.606	0.247
L57	13	4.500	2.740	0.634	0.578	0.799	1.093	-0.097 ^***^	0.207	0.277	0.652	0.472
L64	20	5.950	3.849	0.811	0.685	0.857	1.406	-0.184 ^***^	0.054	0.201	0.994	0.442
Mean	14.786	4.214	2.918	0.644	0.568	0.791	1.076	-0.146 ^***^	0.170	0.281	0.719	0.457

*Nt*, number of total alleles; *Na*, number of observed alleles; *Ne*, number of effective alleles; *Ho*, observed heterozygosity; *He*, expected heterozygosity; *Ht*, total heterozygosity; *I*, Shannon’s information index; *Fis*, inbreeding coefficient within population; *Fit*, inbreeding coefficient across all populations; *Fst*, proportion of differentiation among populations; *F′st*, standardised *Fst* (*Fst/Fstmax*); *Nm*, gene flow. Significant deviation from Hardy–Weinberg expectation is indicated by ***p <0.001.

In terms of genetic diversity at the population level, *Nt* per population ranged from 32 (DL and YD) to 116 (NPA2), whereas *Na* and *Ne* per population ranged from 2.286 (YD) to 7.786 (PXH1) and from 1.759 (DL) to 4.880 (PXH1), respectively (**Table 3**). *Ho* and *He* ranged from 0.465 (LD2) to 0.857 (DPV), and from 0.353 (DL) to 0.739 (PXH1), respectively. Private alleles were detected at 13 of the 20 populations, with the exceptions being WX2, DL, DLH, JF and LD2 on Hainan Island, and DPV and PXH2 in Laos. The inbreeding coefficient, *Fis*, was positive for LD1, LD2 and PXH1, indicating a deficit of heterozygosity at these populations, possibly due to inbreeding. The 11 mainland Indochinese populations had higher mean genetic diversity and more private alleles than did the nine Hainan Island populations, with *He* = 0.599 vs. 0.531, *I* = 1.137 vs. 1.002 and *Np* = 3.636 vs. 0.889, respectively (**Table 3**).

**Table 3:** Genetic diversity metrics by population.

Region	Population	*Nt*	*Np*	*Na*	*Ne*	*Ho*	*He*	*I*	*Fis*	*PPL*
Hainan Island (HN)	WX1	86	2	6.143	3.334	0.636	0.625	1.287	-0.029***	100.00
	WX2	75	0	5.357	3.312	0.658	0.648	1.301	-0.028***	100.00
	WX3	79	2	5.643	3.207	0.656	0.646	1.290	-0.029***	100.00
	YJ	51	1	3.643	2.580	0.613	0.500	0.935	-0.209***	85.71
	DL	32	0	2.286	1.759	0.529	0.353	0.580	-0.449 ns	78.57
	DLH	37	0	2.643	2.138	0.614	0.504	0.806	-0.176 ns	100.00
	JF	40	0	2.857	2.161	0.524	0.471	0.804	-0.099 ns	85.71
	LD1	65	3	4.643	2.956	0.550	0.556	1.108	0.040***	100.00
	LD2	51	0	3.643	2.418	0.465	0.479	0.904	0.000***	85.71
	Mean	57.333	0.889	4.095	2.652	0.583	0.531	1.002	-0.109	92.857
Indochinese mainland (IC)	YD	32	2	2.286	2.081	0.500	0.464	0.739	-0.078 ns	85.71
	PKK	75	6	5.357	3.692	0.786	0.689	1.373	-0.138***	100.00
	DPV	37	0	2.929	2.810	0.857	0.563	0.965	-0.556 ns	85.71
	NPA1	50	4	3.214	2.505	0.714	0.542	0.955	-0.302 ns	92.86
	NPA2	116	9	5.786	2.867	0.825	0.623	1.196	-0.336***	100.00
	PXH1	114	5	7.786	4.880	0.659	0.739	1.665	0.085***	100.00
	PXH2	62	0	3.357	2.425	0.531	0.515	0.927	-0.023 ns	92.86
	XP	58	1	3.714	3.114	0.661	0.607	1.135	-0.118 ns	92.86
	PHR	75	2	4.500	3.361	0.714	0.601	1.182	-0.209**	85.71
	PR	74	9	5.571	3.998	0.667	0.662	1.370	-0.015***	100.00
	KRS	41	2	2.929	2.752	0.714	0.580	0.996	-0.241 ns	92.86
	Mean	66.727	3.636	4.312	3.135	0.693	0.599	1.137	-0.175	93.506
	Overall Mean	62.500	2.000	4.214	2.918	0.644	0.568	1.076	-0.146	93.214

*Nt*, number of total alleles; *Np*, number of private alleles; *Na*, number of observed alleles; *Ne*, number of effective alleles; *Ho*, observed heterozygosity; *He*, expected heterozygosity; *I*, Shannon’s information index; *Fis*, inbreeding coefficient within population; *PPL*, proportion of polymorphism loci. Significant deviation from Hardy–Weinberg expectation is indicated by ***p <0.001 or **p <0.01; ns, not significant.

### 3.2 Genetic differentiation and genetic structure

The inbreeding coefficient per locus within populations (*Fis*) ranged from -0.650 (L53) to 0.054 (L22), with a mean of -0.146, indicating an overall excess of heterozygosity (**Table 2**). Further, the inbreeding coefficient per locus across all populations (*Fit*) ranged from -0.340 (L53) to 0.491 (L51), with a mean value of 0.170. Genetic differentiation per locus across all populations (*Fst*) was positive at all loci with a mean value of 0.281, whereas standardised *Fst* (*F′st*) ranged from 0.230 (L53) to 0.650 (L29), with a mean value of 0.457. Gene flow (*Nm*) ranged from 0.229 (L51) to 1.080 (L53), with a mean value of 0.719 (**Table 2**). AMOVA revealed most genetic variation to lie within populations (81.04%), as opposed to among them (18.96%), and that 10.29% of total genetic variation was accounted for by segregation between Hainan Island (HN) and mainland Indochinese (IC) populations (**Table 4**).

**Table 4:** Results of AMOVA analysis.

Source of genetic variation		*df*	Sum of squares	Variance components	Percentage of variation
Among populations	Between regions (HN and IC)	1	207.469	0.587	10.29
	Within regions	18	335.754	0.495	8.67
	Among populations within regions	303	1348.359	4.450	0.00
Within populations		323	1494.500	4.627	81.04
Total		645	3386.082	5.709	100.00

**Figure 2:**
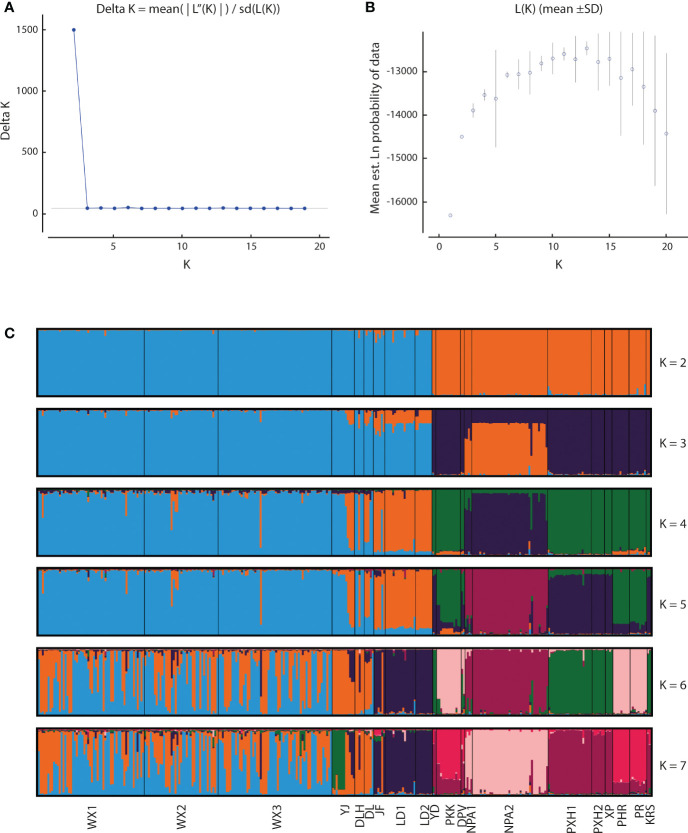
Bayesian estimates of genetic structure in the 20 *Phalaenopsis pulcherrima* populations using STRUCTURE. **(A)** Delta K values identified using STRUCTURE HARVESTER, revealing a peak in Delta K at K = 2. **(B)** Mean likelihood L(K) values (± SD) identified using STRUCTURE. **(C)** Plots of posterior probabilities for 323 individuals assigned to K genetic clusters based on admixture analysis for K = 2 to 7. Different colour bars indicate assignment to different K genetic clusters. Populations are delimited by black lines, with the corresponding population code shown at the bottom.

**Figure 3:**
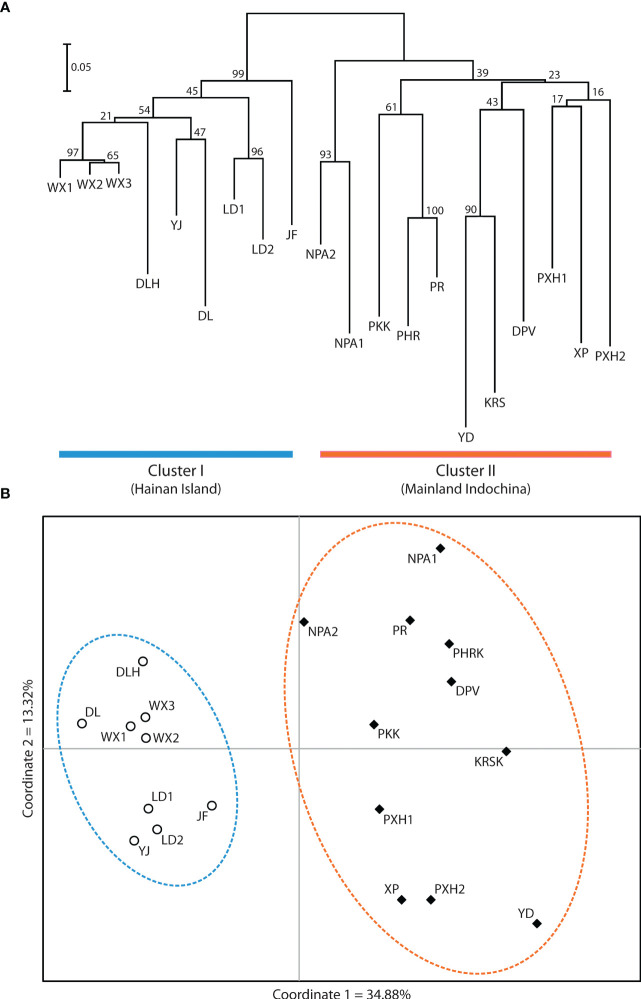
Relatedness among the 20 *Phalaenopsis pulcherrima* populations. **(A)** Relatedness inferred from neighbour-joining analysis of Nei’s genetic distances. Cluster I (blue) contains all nine populations located on Hainan Island, whereas Cluster II (orange) contains all 11 populations located in mainland Indochina. **(B)** Relatedness inferred from PCoA of pairwise genetic distance estimates. The same two clusters are resolved (the nine Hainan Island populations are indicated with open circles and a blue dashed line, and the 11 mainland Indochinese populations are indicated with filled diamonds and an orange dashed line).

**Figure 4:**
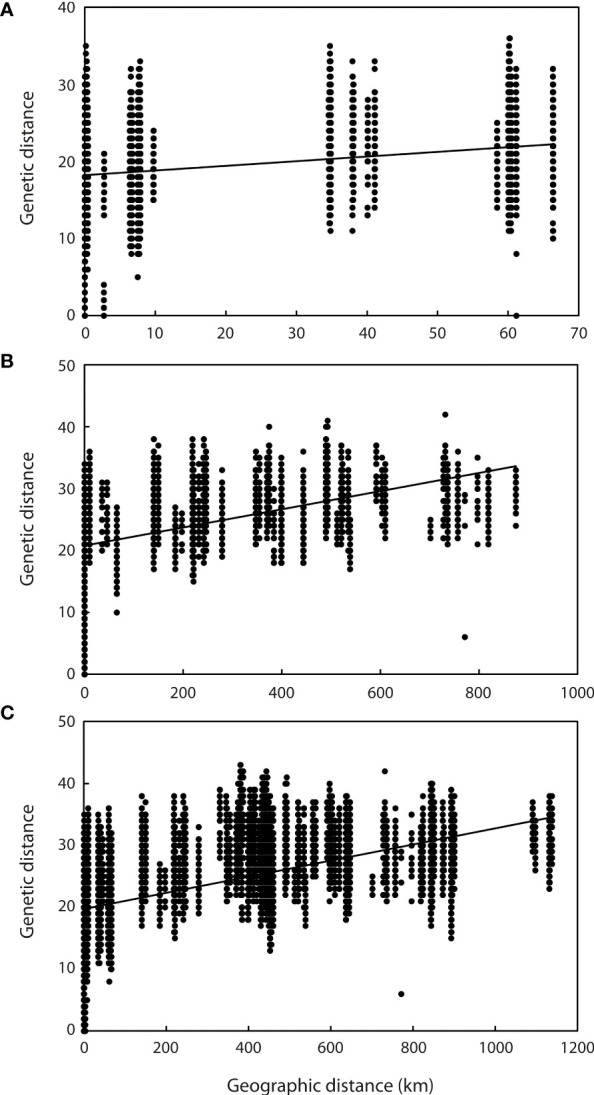
Relationship between genetic and geographic distance among samples. **(A)** Relationship between all samples from the nine Hainan Island populations (*y* = 0.0603*x* + 18.209, *r* = 0.351, *p <*0.001). **(B)** Relationship between all samples from the 11 mainland Indochinese populations (*y* = 0.0147*x* + 20.751, *r* = 0.465, *p <*0.001). **(C)** Relationship between all samples from all 20 populations (*y* = 0.013*x* + 19.724, *r* = 0.625, *p <*0.001).

Application of the LOCPRIOR model in STRUCTURE HARVESTER assigned the highest peak to a K value of 2, with Delta K = 1468.93 (**Figures 2A, B**), indicating that all 323 individuals can be differentiated into two distinct genetic clusters (**Figure 2C**), one containing the nine Hainan Island populations and the other containing the 11 mainland Indochinese populations (**Supplementary File S3**). The same two clearly defined clusters were supported by both NJ analysis (**Figure 3A**) and PCoA (**Figure 3B**). The Mantel test demonstrated a correlation between genetic distance and geographic distance across these two regions, with *r* = 0.351 (*p <*0.001; **Figure 4A**) among all Hainan Island individuals and *r* = 0.465 (*p <*0.001; **Figure 4B**) among all Indochinese individuals. For all individuals across all 20 populations, *r* = 0.625 (*p <*0.001; **Figure 4C**).

### 3.3 Gene flow pattern and population demographic history

The best-fit TPM model detected evidence of a significant bottleneck in both the HN group (*p* = 0.049) and the IC group (*p* = 0.025; **Supplementary File S4**). Evidence of a significant bottleneck was also detected in five populations: WX2 (*p* = 0.017), WX3 (*p* = 0.030), PKK (*p* = 0.005), XP (*p* = 0.048) and PHR (*p* = 0.008).

**Figure 5:**
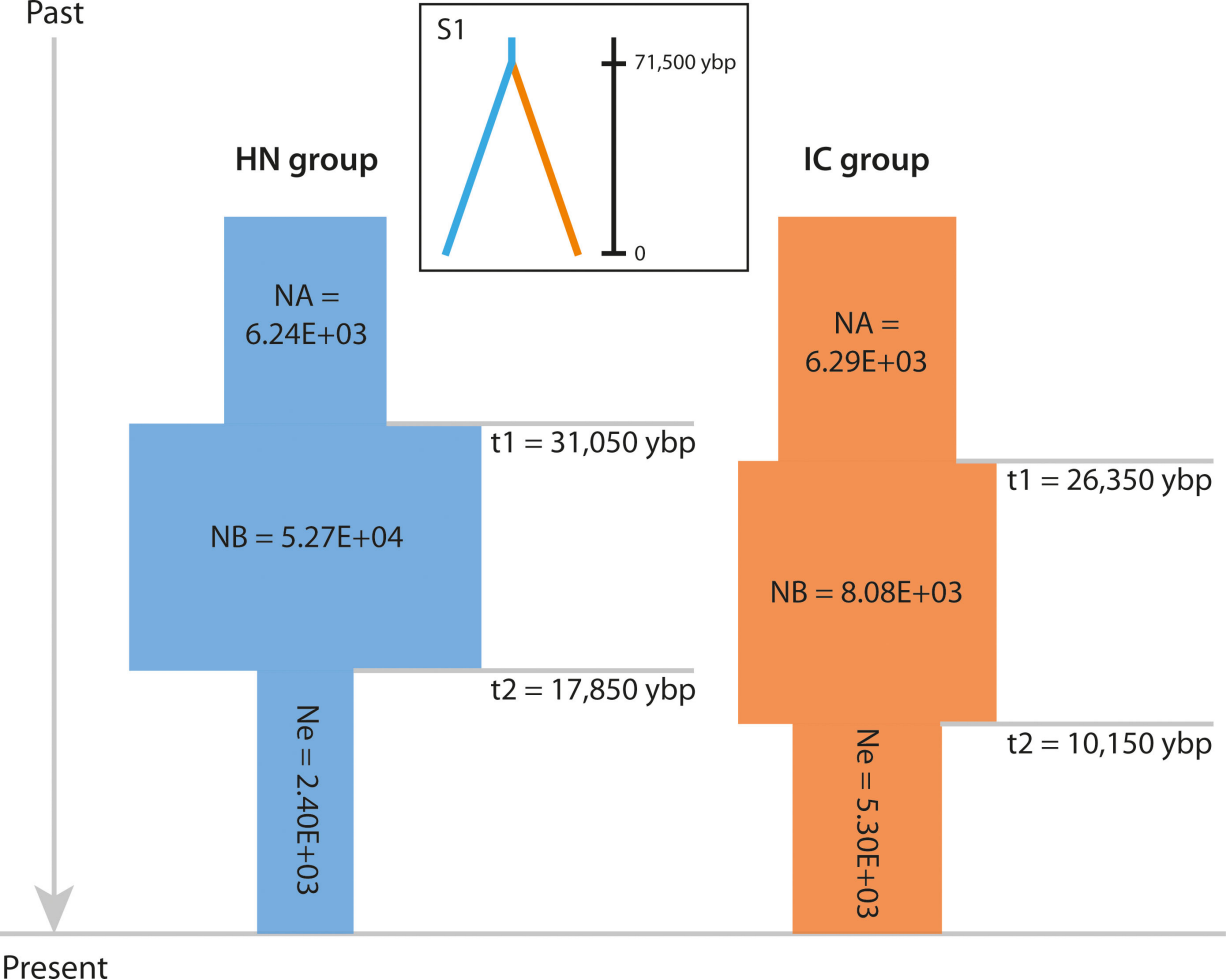
Schematic representation of the demographic history of the HN and IC groups under the best-fit ABC model. NA indicates large ancestral population size, NB indicates expanded historic population size, and Ne indicates current population size. Times of population size changes are indicated by horizontal grey lines and population size estimates (number of individuals) are shown in each block. Best-fit divergence scenario S1 is shown inset. Ybp, years before present.

MIGRATE analysis found model M4 (IC populations are offspring of HN populations with no gene flow between them) to be the most probable (model probability = 1.00; **Supplementary File S5**). Further, DIYABC estimation of divergence history supported scenario S1 (IC group originated from HN group and diverged at t1) as the most likely, with a posterior probability of 0.8736 (95% CI: 0.8623–0.8849; **Supplementary File S2A**). HN group was thus inferred to be ancestral, with subsequent formation and expansion of IC group. These two groups were estimated to have diverged ca. 71,500 years before present (95% CI: 50,500–131,000 ybp; **Figure 5**).

Of the nine potential demographic scenarios, DS1 (expansion + contraction model, Ne < NA) was found to best explain the population history of both HN group (PP = 0.9540, 95% CI: 0.9460–0.9620) and IC group (PP = 0.8126, 95% CI: 0.7824–0.8428; **Supplementary Files S2D**, **S6**). Logistic regression analysis also indicated that both groups had undergone bottlenecks (**Figure 5** and **Supplementary Files S2B**, **S6**), corroborating the evidence provided by the TPM model. Specifically, the ancestral HN group was inferred to have expanded in distribution ca. 31,050 ybp (95% CI: 8,750–44,500 ybp), with its size increasing from 6.24E+03 individuals (95% CI: 1.84E+03–9.82E+03) to 5.27E+04 individuals (95% CI: 8.68E+03–9.76E+04; **Figure 5** and **Supplementary File S6**). A notable bottleneck was inferred ca. 17,850 ybp (95% CI: 3,220–38,050 ybp), involving contraction to 2.40E+03 individuals (95% CI: 8.50E+02–6.15E+03). The derived IC group was inferred to have undergone a similar demographic history after diverging from the ancestral lineage, with expansion from 6.29E+03 individuals (95% CI: 3.30E+03–8.78E+03) to 8.08E+03 individuals (95% CI: 4.82E+03–9.92E+03) ca. 26,350 ybp (95% CI: 5,050–44,050 ybp; **Figure 5** and **Supplementary File S6**). It then experienced a notable bottleneck ca. 10,150 ybp (95% CI: 276.5–24,000 years ago), involving contraction to 5.30E+03 individuals (95% CI: 2.53E+03–8.15E+03).

### 3.4 Fine-scale genetic structure and dispersal estimates

**Figure 6:**
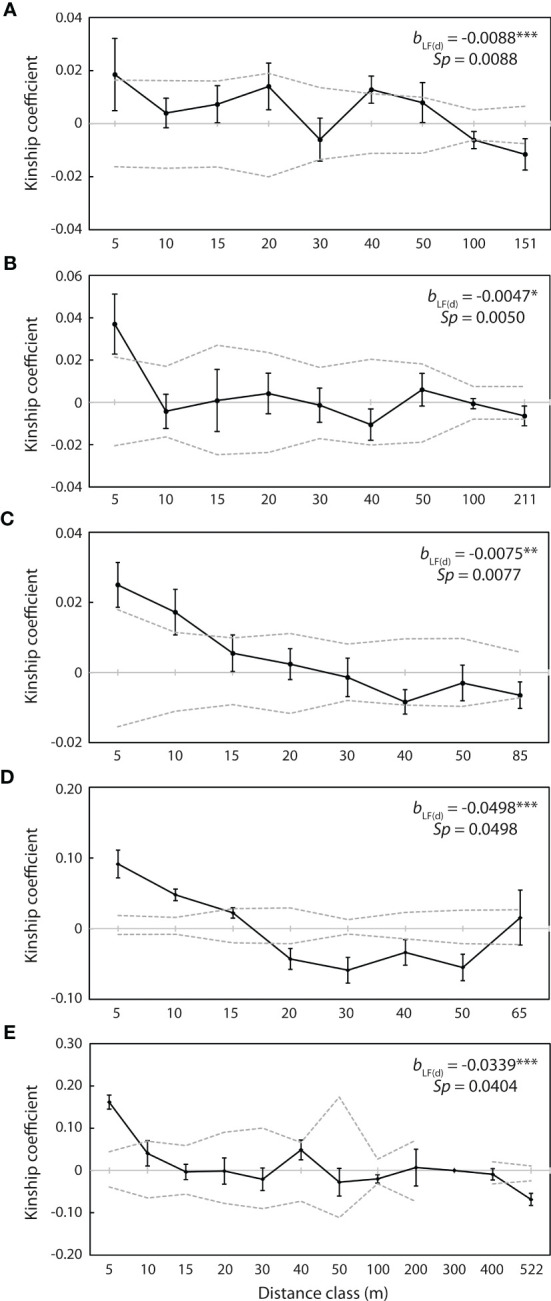
Correlograms of kinship coefficients (*F_ij_*) for individuals at the five populations with more than 20 individuals. **(A)** WX1 (n = 56). **(B)** WX2 (n = 39). **(C)** WX3 (n = 60). **(D)** NPA2 (n = 40). **(E)** PXH1 (n = 23). Closed circles indicate mean co-ancestry values at each distance class. Dashed lines represent upper and lower 95% confidence envelopes around the null hypothesis of no genetic structure [*F_ij_ (d)* = 0]. b_LF_(*d*) represents the slope of the regression of kinship coefficient values, *F_ij_* (*d*), against log distance interval (*d*). Significance is indicated as ***p <0.001, **p <0.01, *p <0.05.

Spatial auto-correlation analysis revealed significant positive genetic kinship (*p <*0.05) over the first 5 m at each of the five populations with more than 20 individuals, with *F_ij_* = 0.019 at WX1, *F_ij_* = 0.037 at WX2, *F_ij_* = 0.025 at WX3, *F_ij_* = 0.092 at NPA2 and *F_ij_* = 0.162 at PXH1 (**Figure 6**). Kinship over the subsequent 5 m was also significantly positive at WX3 (*F_ij_* = 0.017; **Figure 6C**) and NPA2 (*F_ij_* = 0.048; **Figure 6D**), and significant positive kinship was detected at WX1 at the 40 m distance class (*F_ij_* = 0.013; **Figure 6A**). Conversely, significant negative kinship (*p <*0.05) was detected at the 100 m (*F_ij_* = -0.006) and final distance classes (151 m; *F_ij_* = -0.012) at WX1 (**Figure 6A**), at the 20 m (*F_ij_* = -0.043), 30 m (*F_ij_* = -0.059), 40 m (*F_ij_* = -0.034) and 50 m (*F_ij_* = -0.055) distance classes at NPA2 (**Figure 6D**), and at the final distance class (522 m; *F_ij_* = -0.069) at PXH1 (**Figure 6E**).

The slope of the linear regression between *F_ij_* (*d*) and logarithm of geographical distance was significantly negative (*p <*0.05) for all five of these populations, with *b*_LF(_ *_d_*_)_ = -0.0088 at WX1, -0.0047 at WX2, -0.0075 at WX3, -0.0498 at NPA2 and -0.0339 at PXH1 (**Figure 6**). The *Sp* statistic suggested that the intensity of fine-scale genetic structuring declined in the order NPA2 (0.0498) > PXH1 (0.0404) > WX1 (0.0088) > WX3 (0.0077) > WX2 (0.0050).

**Figure 7:**
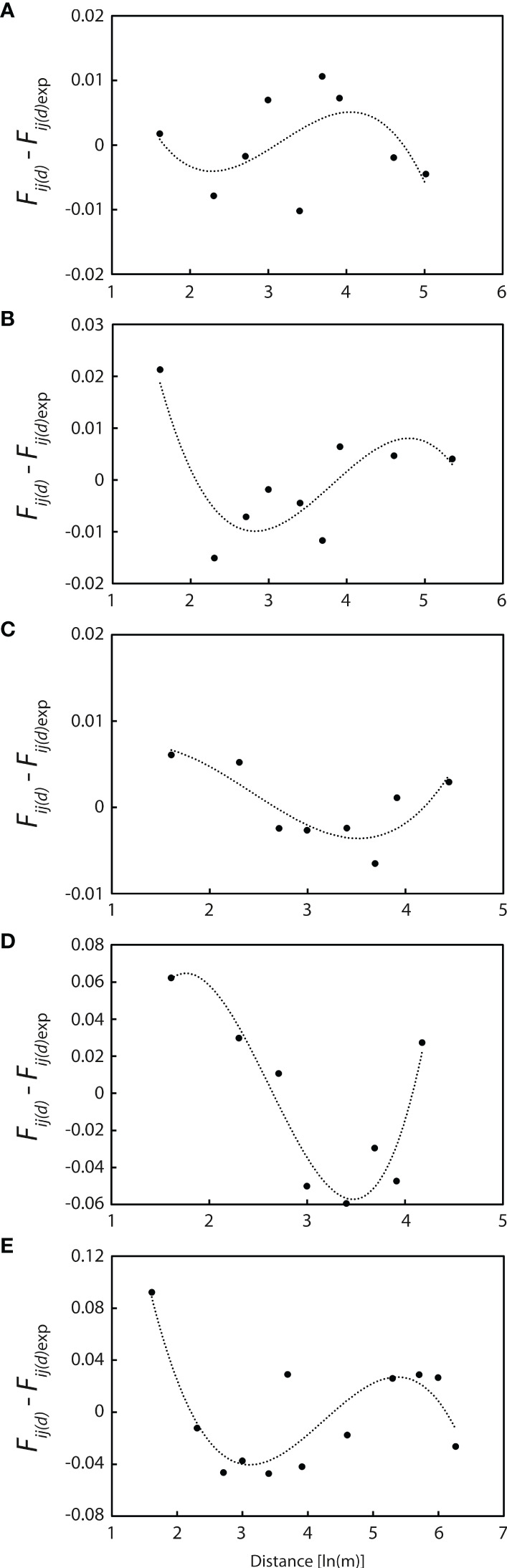
Polynomial regression curves of the third power of residuals [*F_ij (_d_)_*- *F_ij (_d_)_*_exp_] on ln (*d*) for the five populations with more than 20 individuals. **(A)** WX1 (n = 56). **(B)** WX2 (n = 39). **(C)** WX3 (n = 60). **(D)** NPA2 (n = 40). **(E)** PXH1 (n = 23). All curves are concave at short log distances, indicating that gene flow *via* seed is more restricted than that *via* pollen.

Polynomial regression curves of third-power residuals [*F_ij_* (*d*) - *F_ij_* (*d*)_exp_] on ln(*d*) for these five populations are shown in **Figure 7**. All regression lines are concave over shorter distances, with the second derivative *k* = 0.0416 at WX1, 0.0792 at WX2, 0.0073 at WX3, 0.2065 at NPA2 and 0.2402 at PXH1 (**Figure 7**), indicating that seed dispersal is more restricted than pollen dispersal (σ_s_ ≪ σ_p_) at distances of <10 m.

## 4 Discussion

Our analysis of spatial genetic structuring and gene flow in 20 widespread but ecologically confined populations of *Phalaenopsis pulcherrima* reveals high genotypic diversity (*Nt* = 14.786; *Na* = 4.214; *Ne* = 2.918) and high heterozygosity (*Ho* = 0.644; *He* = 0.568) at the species level, and low rates of inbreeding in all but three populations (*Fis* = -0.146). Across a huge swathe of the species’ range in tropical Asia, we uncovered strong evidence for the occurrence of two genetic clusters, one comprising the populations of mainland Indochina and the other those of Hainan Island, with values for a number of genetic diversity metrics (*Nt*, *Np*, *Na*, *Ne*, *Ho*, *He* and *I*) being higher in the former. However, whilst the presence of significantly more variation within populations than between them indicates clear genetic structuring, gene flow was not negligible, with greater variation between the clusters of mainland Indochina and Hainan Island than within them, as well as moderate values for the fixation index (*Fst* = 0.281), standardised *Fst* (*F′st* = 0.457) and proportion of migrants (*Nm* = 0.719), suggesting a degree of interbreeding and migration at the landscape level. Neither fully conforming to, nor fully contradicting, a putative island syndrome, this genetic profile differs from that of many terrestrial habitat island plants studied to date. As such, an explanation for how *P. pulcherrima* balances habitat specificity with ecological connectivity at different geographic scales – and across different matrix types – necessitates a synthesis of the species’ biogeographic and demographic history, as much as an integrated view of its functional ecology.

### 4.1 An array of functional traits underpin compliance with spatial constraints

Inselberg specialists belonging to diverse families including Bromeliacae, Gesneriaceae, Linderniaceae, Orchidaceae and Proteaceae have been shown to exhibit a genetic signature reflecting high divergence between outcrops, limited gene flow at a range of spatial scales and low diversity at the population level (Barbará et al., 2008; Hmeljevski et al., 2014; Pinheiro et al., 2014; Gao et al., 2015; Nistelberger et al., 2015; Lexer et al., 2016; Wanderley et al., 2018). Unifying these taxonomically disparate plants, and underlying their ecological specialisation, are a number of functional traits, including a variable combination of pollinator specificity, self-compatibility, a tendency towards selfing, frequent inbreeding among related individuals and/or short-range seed dispersal. In fact, these traits have been identified in a number of plants restricted to a variety of terrestrial island-like systems, and in some cases they have been linked to clonality and low reproductive success, too (Lhuillier et al., 2006; Lozada-Gobilard et al., 2021). Combined with high habitat specificity, these attributes can render habitat island species prone to fixation and drift (Pinheiro et al., 2014; Nistelberger et al., 2015). On the other hand, a few studies have uncovered evidence of contrasting ecological strategies whereby high rates of outcrossing and at least some landscape-level gene flow are maintained despite natural habitat fragmentation (Heilmeier et al., 2014; Gonçalves-Oliveira et al., 2017), suggesting that long-range dispersal – *via* pollen, seed, or both – is key (Auffret et al., 2017; Jimenez et al., 2017).

As is typical of many inselberg specialists, *P. pulcherrima* is both strongly clonal and has a highly specific mode of pollination characterised by generalised food-deception of solitary bees (*Amegilla zonata* and *Nomia punctulata*), which are attracted by any of a number of co-flowering, rewarding, magnet plants whose flowers have overlapping reflectance spectra in bee colour space (Gale et al., 2019). This mating strategy has been demonstrated not only to reduce geitonogamy and increase xenogamy (Cozzolino and Widmer, 2005), but also promote long-range pollen flow over hundreds of metres and possibly farther (Zhang et al., 2019). Challenging expectations of terrestrial habitat island biogeography (Ottaviani et al., 2020), this can ensure ecologically isolated populations remain genetically interconnected (Zhang et al., 2019). Moreover, in contrast to many other terrestrial habitat island specialists, *P. pulcherrima* is incompletely self-sterile, with self-pollination leading to abortive embryogenesis and significantly reduced seed-set; most recruitment therefore occur as a result of outcrossing (Zhang et al., 2019). This underpins the high genetic diversity, high heterozygosity and low differentiation observed among adjacent populations previously studied on a single large inselberg (Zhang et al., 2019), and probably contributes to the diversity and structuring patterns uncovered here across multiple outcrops.

Fine-scale structure was detected in *P. pulcherrima*, with significant positive kinship consistently detected over the first 5 m interval at each of the five largest populations, and at two of them over the first 10 m, corroborating the findings of Zhang et al. (2019). Further, gene flow estimates revealed seed dispersal to be more restricted than pollen dispersal over equivalent distances (<10 m), suggesting that, over and above clonal propagation, seed dispersal is responsible for the accumulation of related individuals at the microsite level. This reflects the findings of other studies that have reported restricted gene flow *via* orchid seed, despite their minute size and apparent potential for long-distance movement by wind (Jersáková and Malinová, 2007; McCormick and Jacquemyn, 2014; Pinheiro et al., 2014). Although prior research in Hainan revealed relatively low values for the *Sp* statistic (Zhang et al., 2019), *Sp* values derived here (0.0050–0.0498) indicate greater consistency with those reported for several other outcrossing orchids (Jacquemyn et al., 2006; Juárez et al., 2011; Gigant et al., 2016; Sujii et al., 2019), as well as with values published for a range of other outcrossing, wind-dispersed plant taxa, including trees (Vekemans and Hardy, 2004; Piotti et al., 2013; Soliani et al., 2016). At the scale of the populations examined here, this may reflect a rapid drop-off in detectable relatedness at distance classes above 5–10 m due to overlapping seed and pollen movement among multiple, highly heterozygotic individuals, thereby masking any fine-scale spatial genetic patterning, as Sujii et al. (2019) inferred for neotropical inselberg-dwelling *Epidendrum* orchids. Indeed, the detection of significant positive kinship again at the 40 m distance class at one population (WX1) suggests that seed may well travel farther at the site level, with significant negative kinship in general being detected only over much larger distances. In *P. pulcherrima*, a combination of self-sterility, pollination by deceit and high seed motility therefore seem important in promoting panmixia and expanding adaptive possibilities within the constraints of an ecologically confined environment.

### 4.2 Landscape permeability is low but much greater than over water

The Mantel test revealed significant correlation between genetic distance and geographic distance across all 20 populations. With >1,100 km separating the two most distant populations (KRS in Cambodia and DL in China), however, such a relationship is not surprising: Philips et al. (2012) found significant IBD structuring to increase with spatial scale, especially at distances of 250 km and above, among a taxonomically and ecologically diverse array of orchids. The relationship within each of the two highly supported genetic clusters was less pronounced but still significant, with ca. 60 km and ca. 850 km separating the two most distant populations in the HN and IC groups, respectively. This suggests that geographic distance is a key factor in generating the genetic differentiation observed.

Isolation by environment can also impose significant genetic differentiation (Liu et al., 2013; Wang and Bradburd, 2014), even in relatively mobile species and over much shorter geographic distances than those considered here (Mallet et al., 2014). Our estimates of number of migrants per generation (*Nm*) between populations throughout the region ranged from 0.229–1.080 and the mean was less than 1, suggesting that genetic divergence is marked and could become progressively entrenched (Luo et al., 2019). However, whilst low compared with other similarly widespread (but more generalist) orchids (Simmons et al., 2017; Tikendra et al., 2021), these values are comparable to or even higher than those calculated for several other terrestrial habitat specialists (Rossetto et al., 2008; Mallet et al., 2014; Nistelberger et al., 2015; Lexer et al., 2016). Moreover, *Nm* estimates were significantly higher among populations belonging to each of the two genetic clusters (*Nm* = 1.295 for HN group, *Nm* = 0.961 for IC group) than the mean for the species as a whole, suggesting greater gene flow at the scale of either landmass. Similarly, whilst our *Fst* estimate for *P. pulcherrima* is somewhat higher than the mean calculated for the orchid family as a whole (0.146; Philips et al., 2012), it is considerably lower than for many other terrestrial habitat island specialists (e.g., Pinheiro et al., 2014; Gao et al., 2015; Nistelberger et al., 2015; Lexer et al., 2016; Wanderley et al., 2018; Lozada-Gobilard et al., 2021), suggesting that the species is comparatively mobile in the context of habitat specificity.

Despite limited gene flow among inselbergs, the results of AMOVA, STRUCTURE, PCoA and NJ analysis reveal greater connectivity within the Indochinese mainland and Hainan Island than between them, indicating that, for *P. pulcherrima*, terrestrial landscapes are more permeable than open water. This underscores that matrix permeability is indeed important in shaping adaptative specialisation to confined habitats (Ottaviani et al., 2020), with isolated inselbergs scattered across a landscape more likely to serve as ‘stepping stones’ in facilitating regional spread than true islands, even if geographic separation is greater. Accordingly, in possessing a combination of traits that promote landscape-scale gene flow (deceptive pollination and wind-borne seed dispersal) on the one hand, as well as site-level persistence (clonality, incomplete self-sterility and an ability to take advantage of multiple magnet species) on the other, *P. pulcherrima* appears to be better able to ensure functional connectivity (Auffret et al., 2017) than might be predicted from the perspective of a putative island syndrome (Itescu, 2019; Ottaviani et al., 2020) and as borne out by various ‘more typical’ terrestrial habitat island plants (Rossetto et al., 2008; Hmeljevski et al., 2014; Gao et al., 2015).

### 4.3 Demographic history sheds light on temporal aspects of divergence and spread

Biogeography is also an important factor shaping genetic and demographic trajectories (Riddle et al., 2008). In light of assorted geo-physical and biological evidence, Hainan Island is thought to have been intermittently connected to nearby mainland Asia (specifically, the coastline of modern-day Vietnam) *via* the periodic emergence of a land bridge during Quaternary glacial cycles (Ali, 2018; Lin et al., 2021). The island lies on a continental shelf, presently submerged <50 m below sea level (Ali, 2018), that would have been exposed during cooler, drier periods, including throughout much of the time from the close of the last interglacial ca. 120,000 ybp until the end of the Last Glacial Maximum ca. 20,000 ybp (Bintanja et al., 2005). It is hypothesised that ancestors of Hainan Island’s present-day flora would have migrated in from adjacent areas during such intervals (Ali, 2018); indeed, Lin et al. (2021) report strong nestedness in relation to the flora of Vietnam. This correlates closely with the divergence history inferred here for *P. pulcherrima* based on DIYABC analysis, with separation of the IC and HN groups estimated at ca. 71,500 ybp.

Intriguingly, however, our results provide unequivocal support for the HN group as ancestral and the IC group as derived, with each group since experiencing independent, though similar, demographic histories characterised by an absence of gene flow between them (MIGRATE model probability = 1.00; DIYABC posterior probability 0.8736). Thus, a demographic scenario involving expansion and then abrupt contraction was found to explain the population history of both groups, with broad synchronicity as well as a consistent lag of a few thousand years in the IC group relative to the HN group in the timing of these events, being suggestive of common forcing factors, potentially climatic. Not only does logistic regression corroborate evidence of corresponding, regional declines provided by the TPM model, but these analyses also confirm the marked isolation of populations either side of today’s Gulf of Tonkin as revealed by STRUCTURE and other clustering approaches. The divergence of island and mainland lineages in *P. pulcherrima* thus appears to be recent but genetically prominent nevertheless, with isolation at the species level being imposed predominantly by sea-level rise, rather than by the presence of unsuitable intervening terrestrial habitat. Indeed, expansion of both groups subsequent to divergence suggests that biogeographic spread took place within either landmass as a result of migration over the past ca. 30,000 years, implicating landscape-level gene flow across matrix vegetation types: we detected a genetic bottleneck signature in five populations, pointing to a history of independent, long-range colonisation events in a quarter of all populations on both landmasses. Meanwhile, the inferred direction of gene flow – from island to mainland – adds weight to the view posited by Hutsemékers et al. (2011) that island populations can serve as refugia for postglacial colonisation of continental landmasses by effective dispersers.

### 4.4 Island syndromes: Not a singular picture

By combining strong clonality with self-sterility, and generalised food-deception of specific pollinators with wind-borne seed dispersal, *P. pulcherrima* exhibits several features typical of terrestrial habitat island plants but not the full suite of functional traits predicted by theoretical framing of a putative island syndrome. Within the constraints of eco-evolutionary trade-offs for on-spot persistence and landscape-scale connectivity, it therefore appears that life in terrestrial habitat fragments drives multitudinous adaptive strategies, not fixed responses. Mirroring the course of debate on the existence of syndromes in other areas of ecological theory, such as pollination (Dellinger, 2020) and plant defense (Agrawal, 2007), identifying common principles and caveats for the application of island biogeographic theories to terrestrial island-like systems therefore seems constructive. We contend that scrutiny of matrix properties in determining permeability at a range of spatial scales will be important (Ottaviani et al., 2020), as will a consideration of the temporal dimension in its capacity to shape demographic trajectories and biogeographic spread.

To our knowledge, this is the first study to focus on the landscape genetics of a specialist of tropical Asia’s granitic inselbergs. Beyond an examination of the link between adaptive characters and gene flow that is relevant to understanding island syndromes, our results highlight the need for more work on this threatened habitat type, both for the conservation of the vulnerable and often endemic species they host as well as the temporo-spatial landscape features they embody (Fitzsimmons and Michael, 2016; Auffret et al., 2017).

## Data availability statement

The original contributions presented in the study are included in the article/**Supplementary Material**, further inquiries can be directed to the corresponding author/s.

## Author contributions

SG conceived and designed the study. SG, JL, SS, SB and LA conducted field work and collected the samples. SG and JL processed the samples. ZZ analyzed the data and generated all statistical outputs. SG wrote the manuscript with input from ZZ and JL. All authors contributed to the article and approved the submitted version.

## References

[B1] Adams B. J. Schnitzer S. A. Yanoviak S. P. (2017). Trees as islands: Canopy ant species richness increases with the size of liana-free trees in a Neotropical forest. Ecography 40, 1067–1075. doi: 10.1111/ecog.02608

[B2] Agrawal A. A. (2007). Macroevolution of plant defense strategies. Trends Ecol. Evol. 22, 103–109. doi: 10.1016/j.tree.2006.10.012 17097760

[B3] Ali J. R. (2018). New explanation for elements of Hainan Island’s biological assemblage may stretch things a little too far. Ecography 41, 457–460. doi: 10.1111/ecog.03199

[B4] Auffret A. G. Rico Y. Bullock J. M. Hooftman D. A. P. Pakeman R. J. Soons M. B.. (2017). Plant functional connectivity – integrating landscape structure and effective dispersal. J. Ecol. 105, 1648–1656. doi: 10.1111/1365-2745.12742

[B5] Averyanov L. V. (2009). *Doritis pulcherrima* var. *apiculata* (Orchidaceae): A new variety from southern Vietnam and conditions of its natural habitat. Lindleyana 22, 9–16.

[B6] Barbará T. Martinelli G. Palma-Silva C. Fay M. F. Mayo S. Lexer C. (2008). Genetic relationships and variation in reproductive strategies in four closely related bromeliads adapted to neotropical ‘inselbergs’: *Alcantarea glaziouana*, *A. regina*, *A. geniculata* and *A. imperialis* (Bromeliaceae). Ann. Bot. 103, 65–77. doi: 10.1093/aob/mcn226 PMC2707295 19074451

[B7] Beerli P. (2009). “How to use MIGRATE or why Markov chain Monte Carlo programs difficult to use,” in Population genetics for animal conservation, Vol. 17 . Eds. Bertorelle G. Bruford M. W. Hauffe H. C. Rizzoli A. Vernesi C. (Cambridge, UK: Cambridge University Press), 42–79.

[B8] Bintanja R. van de Wal R. S. W. Oerlemans J. (2005). Modelled atmospheric temperatures and global sea levels over the past million years. Nature 437, 125–128. doi: 10.1038/nature03975 16136140

[B9] Burns K. C. (2019). Evolution in isolation: The search for an island syndrome in plants (Cambridge UK: Cambridge University Press).

[B10] Carlquist S. (1974). Island biology (New York, USA: Columbia University Press).

[B11] Chomicki G. Bidel L. P. Ming F. Coiro M. Zhang X. Wang Y.. (2015). The velamen protects photosynthetic orchid roots against UV-b damage, and a large dated phylogeny implies multiple gains and losses of this function during the Cenozoic. New Phytol. 205, 1330–1341. doi: 10.1111/nph.13106 25345817

[B12] Christenson E. A. (2001). Phalaenopsis – a monograph (Portland, US: Timber Press).

[B13] Cornuet J. M. Pudlo P. Veyssier J. Dehne- Garcia A. Gautier M. Leblois R.. (2014). DIYABC v2.0: A software to make approximate Bayesian computation inferences about population history using single nucleotide polymorphism, DNA sequence and microsatellite data. Bioinformatics 30, 1187–1189. doi: 10.1093/bioinformatics/btt763 24389659

[B14] Cornuet J. M. Ravigne V. Estoup A. (2010). Inference on population history and model checking using DNA sequence and microsatellite data with the software DIYABC (v1.0). BMC Bioinf. 11, 401. doi: 10.1186/1471-2105-11-401 PMC2919520 20667077

[B15] Cozzolino S. Widmer A. (2005). Orchid diversity: An evolutionary consequence of deception? Trends Ecol. Evol. 20, 487–494. doi: 10.1016/j.tree.2005.06.004 16701425

[B16] Dellinger A. S. (2020). Pollination syndromes in the 21^st^ century: Where do we stand and where may we go? New Phytol. 228, 1193–1213. doi: 10.1111/nph.16793 33460152

[B17] Di Rienzo A. Peterson A. C. Garza J. C. Valdes A. M. Slatkin M. Freimer N. B. (1994). Mutational processes of simple-sequence repeat loci in human populations. Proc. Natl. Acad. Sci. 91, 3166–3170. doi: 10.1073/pnas.91.8.3166 8159720 PMC43536

[B18] Earl D. A. vonHoldt B. M. (2012). STRUCTURE HARVESTER: A website and program for visualizing STRUCTURE output and implementing the evanno method. Conserv. Genet. Resour. 4, 359–361. doi: 10.1007/s12686-011-9548-7

[B19] Evanno G. Regnaut S. Goudet J. (2005). Detecting the number of clusters of individuals using the software STRUCTURE: A simulation study. Mol. Ecol. 14, 2611–2620. doi: 10.1111/j.1365-294X.2005.02553.x 15969739

[B20] Fine P. V. Baraloto C. (2016). Habitat endemism in white-sand forests: Insights into the mechanisms of lineage diversification and community assembly of the Neotropical flora. Biotropica 48, 24–33. doi: 10.1111/btp.12301

[B21] Fitzsimmons J. A. Michael D. R. (2016). Rocky outcrops: A hard road in the conservation of critical habitats. Biol. Conserv. 211, 36–44. doi: 10.1016/j.biocon.2016.11.019

[B22] Gale S. W. Li J. Fischer G. A. (2019). Population ecology, conservation and reintroduction of phalaenopsis pulcherrima (Orchidaceae) in Hainan Province, China. Internal Report. (Hong Kong, China: Kadoorie Farm and Botanic Garden)

[B23] Gao Y. Ai B. Kong H. Kang M. Huang H. (2015). Geographical pattern of isolation and diversification in karst habitat islands: A case study in the *Primulina eburnea* complex. J. Biogeogr. 42, 2131–2144. doi: 10.1111/jbi.12576

[B24] Gigant R. L. de Bruyn A. M’sa T. Viscardi G. Gigord L. Gauvin-Bialecki A.. (2016). Combining pollination ecology and fine-scale spatial genetic structure analysis to unravel the reproductive strategy of an insular threatened orchid. South Afr. J. Bot. 105, 25–35. doi: 10.1016/j.sajb.2016.02.205

[B25] Gomes P. Alves M. (2009). Floristic and vegetational aspects of an inselberg in the semi-arid region of northeast Brazil. Edinburgh. J. Bot. 66, 329–346. doi: 10.1017/S0960428609005241

[B26] Gonçalves-Oliveira R. C. Wöhrmann T. Benko-Iseppon A. M. Krapp F. Alves M. Wanderley M. D. G. L.. (2017). Population genetic structure of the rock outcrop species *Encholirium spectabile* (Bromeliaceae): The role of pollination vs. seed dispersal and evolutionary implications. Am. J. Bot. 104, 868–878. doi: 10.3732/ajb.1600410 28611073

[B27] Goudet J. (1995). FSTAT (version 1.2): A computer program to calculate f-statistics. J. Heredit. 86, 485–486. doi: 10.1093/oxfordjournals.jhered.a111627

[B28] Govaerts R. Bernet P. Kratochvil K. Gerlach G. Carr G. Alrich P.. (2021) World checklist of Orchidaceae. The Board of Trustees of the Royal Botanic Gardens, Kew. Available at: www.kew.org/wcsp/monocots/ (Accessed 18 October 2021).

[B29] Grossenbacher D. L. Brandvain Y. Auld J. R. Burd M. Cheptou P. O. Conner J. K.. (2017). Self-compatibility is over-represented on islands. New Phytol. 215, 469–478. doi: 10.1111/nph.14534 28382619

[B30] Hardy O. J. Vekemans X. (2002). SPAGeDi: A versatile computer program to analyse spatial genetic structure at the individual or population levels. Mol. Ecol. Notes 2, 618–620. doi: 10.1046/j.1471-8286.2002.00305.x

[B31] Hedrick P. W. (2005). A standardized genetic differentiation measure. Evolution 59, 1633–1638. doi: 10.1111/j.0014-3820.2005.tb01814.x 16329237

[B32] Heilmeier H. Hartung W. Durka W. (2014). Habitat conditions, population genetics and niche partitioning of the Namibian resurrection plant *Chamaegigas intrepidus* Dinter. Contributii Botanice 49, 109–120.

[B33] Henneron L. Sarthou C. de Massary J. C. Ponge J. F. (2019). Habitat diversity associated to island size and environmental filtering control the species richness of rock-savanna plants in neotropical inselbergs. Ecography 42, 1536–1547. doi: 10.1111/ecog.04482

[B34] Heuertz M. (2010). Estimating seed vs. pollen dispersal from spatial genetic structure in the common ash. Mol. Ecol. 12, 2483–2495. doi: 10.1046/j.1365-294X.2003.01923.x 12919486

[B35] Hmeljevski K. V. Freitas L. Domingues R. Pereira A. R. Cancio A. S. Andrade A. C. S.. (2014). Conservation assessment of an extremely restricted bromeliad highlights the need for population-based conservation on granitic inselbergs of the Brazilian Atlantic forest. Flora 209, 250–259. doi: 10.1016/j.flora.2014.03.004

[B36] Hu X. Lan S. Song X. Yang F. Zhang Z. Peng D.. (2021). Genetic divergence between two sympatric ecotypes of *Phalaenopsis pulcherrima* on Hainan Island. Diversity 13, 446.

[B37] Hutsemékers V. Szövényi P. Shaw A. J. González-Mancebo J.-M. Muñoz J. Vanderpoorten A. (2011). Oceanic islands are not sinks of biodiversity in spore-producing plants. Proc. Natl. Acad. Sci. 108, 18989–18994. doi: 10.1073/pnas.1109119108 22084108 PMC3223459

[B38] Inuthai J. Sridith K. (2010). The vegetation structure on the granitic inselberg in songkhla province, Peninsular Thailand. Thai. For. Bull. (Botany). 38, 74–89.

[B39] Itescu Y. (2019). Are island-like systems biologically similar to islands? A review of the evidence. Ecography 42, 1298–1314. doi: 10.1111/ecog.03951

[B40] Jacquemyn H. Brys R. Vandepitte K. Honnay O. Roldán-Ruiz I. (2006). Fine-scale genetic structure of life history stages in the food-deceptive orchid *Orchis purpurea* . Mol. Ecol. 15, 2801–2808. doi: 10.1111/j.1365-294X.2006.02978.x 16911201

[B41] Jersáková J. Malinová T. (2007). Spatial aspects of seed dispersal and seedling recruitment in orchids. New Phytol. 176, 237–241. 17888110 10.1111/j.1469-8137.2007.02223.x

[B42] Jimenez J. F. Sánchez-Gómez P. Cánovas J. L. Hensen I. Aouissat M. (2017). Influence of natural habitat fragmentation on the genetic structure of Canarian populations of *Juniperus turbinata* . Silva Fennica 51, 1678. doi: 10.14214/sf.1678

[B43] Jin X. Li D. Ren Z. Xiang X. (2012). A generalized deceptive pollination system of *Doritis pulcherrima* (Aeridinae: Orchidaceae) with non-reconfigured pollinaria. BMC Plant Biol. 12, 67. 22571550 10.1186/1471-2229-12-67 PMC3388949

[B44] Juárez L. Montaña C. Ferrer M. M. (2011). Genetic structure at patch level of the terrestrial orchid *Cyclopogon luteoalbus* (Orchidaceae) in a fragmented cloud forest. Plant Systemat. Evol. 297, 237–251. doi: 10.1007/s00606-011-0511-6

[B45] Kalisz S. Nason J. D. Hanzawa F. M. Tonsor S. J. (2001). Spatial population genetic structure in *Trillium grandiflorum*: The roles of dispersal, mating, history, and selection. Evolution 55, 1560–1568. doi: 10.1111/j.0014-3820.2001.tb00675.x 11580015

[B46] Kluge M. Büdel B. (2009). “Inselbergs: Vegetation, diversity and ecology,” in Tropical biology and conservation management, Vol. 4 . Eds. Del Claro K. Oliveira P. S. Rico-Gray V. (Paris, FR: UNESCO-EOLSS).

[B47] Kumar P. Gale S. W. Pedersen H.Æ. Phaxaysombath T. Bouamanivong S. Fischer G. A. (2018). Additions to the orchid flora of Laos and taxonomic notes on orchids of the Indo-Burma region. Taiwania 63, 61–83.

[B48] Lexer C. Marthaler F. Humbert S. Barbará T. de la Harpe M. Bossolini E.. (2016). Gene flow and diversification in a species complex of *Alcantarea* inselberg bromeliads. Botanical J. Linn. Soc. 181, 505–520. doi: 10.1111/boj.12372

[B49] Lhuillier E. Butaud J.-F. Bouvet J.-M. (2006). Extensive clonality and strong differentiation in the insular Pacific tree *Santalum insulare*: Implications for its conservation. Ann. Bot. 98, 1061–1072. doi: 10.1093/aob/mcl190 16945947 PMC3292246

[B50] Liira J. Jurjendal I. Paal J. (2014). Do forest plants conform to the theory of island biogeography: the case study of bog islands. Biodivers. Conserv. 23, 1019–1039. doi: 10.1007/s10531-014-0650-5

[B51] Lin S. Chen L. Peng W. Yu J. He J. Jiang H. (2021). Temperature and historical land connectivity jointly shape the floristic relationship between Hainan Island and the neighbouring landmasses. Sci. Total. Environ. 769, 144629. doi: 10.1016/j.scitotenv.2020.144629 33477038

[B52] Liu M. Zhang J. Chen Y. Compton S. G. Chen X. Y. (2013). Contrasting genetic responses to population fragmentation in a coevolving fig and fig wasp across a mainland-island archipelago. Mol. Ecol. 22, 4384–4396. doi: 10.1111/mec.12406 23879300

[B53] Loiselle B. A. Sork V. L. Nason J. Graham C. (1995). Spatial genetic structure of a tropical understory shrub, *Psychotria officinalis* (Rubiaceae). Am. J. Bot. 82, 1420–1425. doi: 10.1002/j.1537-2197.1995.tb12679.x

[B54] Lozada-Gobilard S. Schwarzer C. Dyer R. Tiedemann R. Joshi J. (2021). Genetic diversity and connectivity in plant species differing in clonality and dispersal mechanisms in wetland island habitats. J. Heredit. 112, 108–121. doi: 10.1093/jhered/esaa059 33555304

[B55] Luo Z. Brock J. Dyer J. M. Kutchan T. Schachtman D. Augustin M.. (2019). Genetic diversity and population structure of a *Camelina sativa* spring panel. Front. Plant Sci. 10, 184. doi: 10.3389/fpls.2019.00184 30842785 PMC6391347

[B56] MacArthur R. Wilson E. O. (1967). The theory of island biogeography (Princeton, USA: Princeton University Press).

[B57] Mallet B. Martos F. Blambert L. Pailler T. Humeau L. (2014). Evidence for isolation-by-habitat among populations of an epiphytic orchid species on a small oceanic island. PloS One 9, e87469. doi: 10.1371/journal.pone.0087469 24498329 PMC3911949

[B58] McCormick M. K. Jacquemyn H. (2014). What constrains the distribution of orchid populations? New Phytol. 202, 392–400. doi: 10.1111/nph.12639

[B59] Meirmans P. G. (2006). Using the AMOVA framework to estimate a standardized genetic differentiation measure. Evolution 60, 2399–2402. doi: 10.1111/j.0014-3820.2006.tb01874.x 17236430

[B60] Mendez-Castro F. E. Conti L. Chytrý M. Jiménez-Alfaro B. Hájek M. Horsák M.. (2021). What defines insularity for plants in edaphic islands? Ecography 44, 1249–1258. doi: 10.1111/ecog.05650

[B61] Nistelberger H. M. Byrne M. Coates D. Roberts J. D. (2015). Genetic drift drives evolution in the bird-pollinated, terrestrial island endemic *Grevillea georgeana* (Proteaceae). Botanical J. Linn. Soc. 178, 155–168. doi: 10.1111/boj.12270

[B62] Ottaviani G. Keppel G. Götzenberger L. Harrison S. Opedal Ø.H. Conti L.. (2020). Linking plant functional ecology to island biogeography. Trends Plant Sci. 25, 329–339. doi: 10.1016/j.tplants.2019.12.022 31953170

[B63] Peakall R. Smouse P. E. (2012). GenAlEx 6.5: Genetic analysis in Excel. Population genetic software for teaching and research – an update. Bioinformatics 28, 2537–2539. doi: 10.1093/bioinformatics/bts460 22820204 PMC3463245

[B64] Philips R. D. Dizon K. W. Peakall R. (2012). Low population genetic differentiation in the Orchidaceae: Implications for the diversification of the family. Mol. Ecol. 21, 5208–5220. doi: 10.1111/mec.12036 23017205

[B65] Pinheiro F. Cozzolino S. Draper D. de Barros F. Félix L. P. Fay M. F.. (2014). Rock outcrop orchids reveal the genetic connectivity and diversity of inselbergs of northeastern Brazil. BMC Evolution. Biol. 14, 49. doi: 10.1186/1471-2148-14-49 PMC4004418 24629134

[B66] Piotti A. Leonardi S. Heuertz M. Buiteveld J. Geburek T. Gerber S.. (2013). Within-population genetic structure in beech (*Fagus sylvatica* l.) stands characterized by different disturbance histories: Does forest management simplify population substructure? PloS One 8, e73391. doi: 10.1371/journal.pone.0073391 24039930 PMC3764177

[B67] Piry S. Luikart G. Cornuet J. M. (1999). Computer note. BOTTLENECK: A computer program for detecting recent reductions in the effective size using allele frequency data. J. Heredit. 90, 502–503. doi: 10.1093/jhered/90.4.502

[B68] Porembski S. Barthlott W. (2000). Inselbergs – biotic diversity of isolated rock outcrops in tropical and temperate regions (Berlin, Germany: Springer)

[B69] Pritchard J. K. Stephens M. Donnelly P. (2000). Inference of population structure using multilocus genotype data. Genetics 155, 945–959. doi: 10.1093/genetics/155.2.945 10835412 PMC1461096

[B70] Raymond M. Rousset F. (1995). Genepop (Version 1.2) – population-genetics software for exact tests and ecumenicism. J. Heredit. 86, 248–249. doi: 10.1093/oxfordjournals.jhered.a111573

[B71] Riddle B. R. Dawson M. N. Hadly E. A. Hafner D. J. Hickerson M. J. Mantooth S. J.. (2008). The role of molecular genetics in sculpting the future of integrative biogeography. Prog. Phys. Geogr. 32, 173–202.

[B72] Rossetto M. Kooyman R. Sherwin W. Jones R. (2008). Dispersal limitations, rather than bottlenecks or habitat specificity, can restrict the distribution of rare and endemic rainforest trees. Am. J. Bot. 95, 321–329. doi: 10.3732/ajb.95.3.321 21632357

[B73] Simmons L. Mathieson M. T. Lamont R. W. Shapcott A. (2017). Genetic diversity of endangered orchid *Phaius australis* across a fragmented Australian landscape. Conserv. Genet. 19, 451–465. doi: 10.1007/s10592-017-1022-y

[B74] Soliani C. Vendramin G. G. Gallo L. A. Marchelli P. (2016). Logging by selective extraction of best trees: Does it change patterns of genetic diversity? The case of *Nothofagus pumilio* . For. Ecol. Manage. 373, 81–92. doi: 10.1016/j.foreco.2016.04.032

[B75] Sujii P. S. Cozzolino S. Pinheiro F. (2019). Hybridization and geographic distribution shapes the spatial genetic structure of two co-occurring orchid species. Heredity 123, 458–469. doi: 10.1038/s41437-019-0254-7 31391556 PMC6781141

[B76] Takezaki N. Nei M. Tamura K. (2014). POPTREEW: Web version of POPTREE for constructing population trees from allele frequency data and computing some other quantities. Mol. Biol. Evol. 31, 1622–1624. doi: 10.1093/molbev/msu093 24603277

[B77] Tamura K. Stecher G. Peterson D. Filipski A. Kumar S. (2013). MEGA6: Molecular evolutionary genetics analysis version 6.0. Mol. Biol. Evol. 30, 2725–2729. doi: 10.1093/molbev/mst197 24132122 PMC3840312

[B78] Tikendra L. Potshangbam A. M. Amom T. Dey A. Nongdam P. (2021). Understanding the genetic diversity and population structure of *Dendrobium chrysotoxum* Lindl. – an endangered medicinal orchid and implication for its conservation. South Afr. J. Bot. 138, 364–376. doi: 10.1016/j.sajb.2021.01.002

[B79] van Oosterhout C. Hutchinson W. F. Wills D. P. M. Shipley P. (2004). MICRO-CHECKER: Software for identifying and correcting genotyping errors in microsatellite data. Mol. Ecol. Notes 4, 535–538. doi: 10.1111/j.1471-8286.2004.00684.x

[B80] Vekemans X. Hardy O. J. (2004). New insights from fine-scale spatial genetic structure analyses in plant populations. Mol. Ecol. 13, 921–935. doi: 10.1046/j.1365-294X.2004.02076.x 15012766

[B81] Wanderley A. M. Machado I. C. S. de Almeida E. M. Felix L. P. Galetto L. Benko-Iseppon A. M.. (2018). The roles of geography and environment in divergence within and between two closely related plant species inhabiting an island-like habitat. J. Biogeogr. 45, 381–393. doi: 10.1111/jbi.13137

[B82] Wang I. J. Bradburd G. S. (2014). Isolation by environment. Mol. Ecol. 23, 5649–5662. doi: 10.1111/mec.12938 25256562

[B83] Zhang Z. Gale S. W. Li J.-H. Fischer G. A. Ren M.-X. Song X.-Q. (2019). Pollen-mediated gene flow ensures connectivity among spatially discrete sub-populations of *Phalaenopsis pulcherrima*, a tropical food-deceptive orchid. BMC Plant Biol. 19, 597. doi: 10.1186/s12870-019-2179-y 31888488 PMC6937714

[B84] Zhang S. Yang Y. Li J. Qin J. Zhang W. Huang W.. (2018). Physiological diversity of orchids. Plant Diversity 40, 196–208. doi: 10.1016/j.pld.2018.06.003 30740565 PMC6137271

