# Supplementary material for: Exploring island syndromes: Variable matrix permeability in Phalaenopsis pulcherrima (Orchidaceae), a specialist lithophyte of tropical Asian inselbergs
Source: Front Plant Sci. 2023 Feb 20;14:1097113. doi: 10.3389/fpls.2023.1097113 (PMC9986494; doi:10.3389/fpls.2023.1097113)
Supplement: Supplementary file 1 [file Table_1.docx]

Supplementary File S1. Descriptions of prior settings for all parameters used in the DIY-ABC.

| Parameters | Prior distributions |
| --- | --- |
| **Divergence model** | |
| HN group | Uniform[1E1–1E4] |
| IC group | Uniform[1E1–1E4] |
| t | Uniform[1E1–1E4] |
| **Demographic history model** | |
| HN group | |
| Ne | Uniform[1E1–1E4] |
| t2 | Uniform[1E1–9E3] |
| NB | Uniform[1E3–1E5] |
| t1 | Uniform[1E3–1E5] |
| NA | Uniform[1E1–9E3] |
| IC group | |
| Ne | Uniform[1E1–1E4] |
| t2 | Uniform[1E1–5E3] |
| NB | Uniform[1E2–1E4] |
| t1 | Uniform[1E1–9E3] |
| NA | Uniform[1E1–9E3] |
