# Supplementary material for: Exploring island syndromes: Variable matrix permeability in Phalaenopsis pulcherrima (Orchidaceae), a specialist lithophyte of tropical Asian inselbergs
Source: Front Plant Sci. 2023 Feb 20;14:1097113. doi: 10.3389/fpls.2023.1097113 (PMC9986494; doi:10.3389/fpls.2023.1097113)
Supplement: Supplementary file 2 [file Table_2.docx]

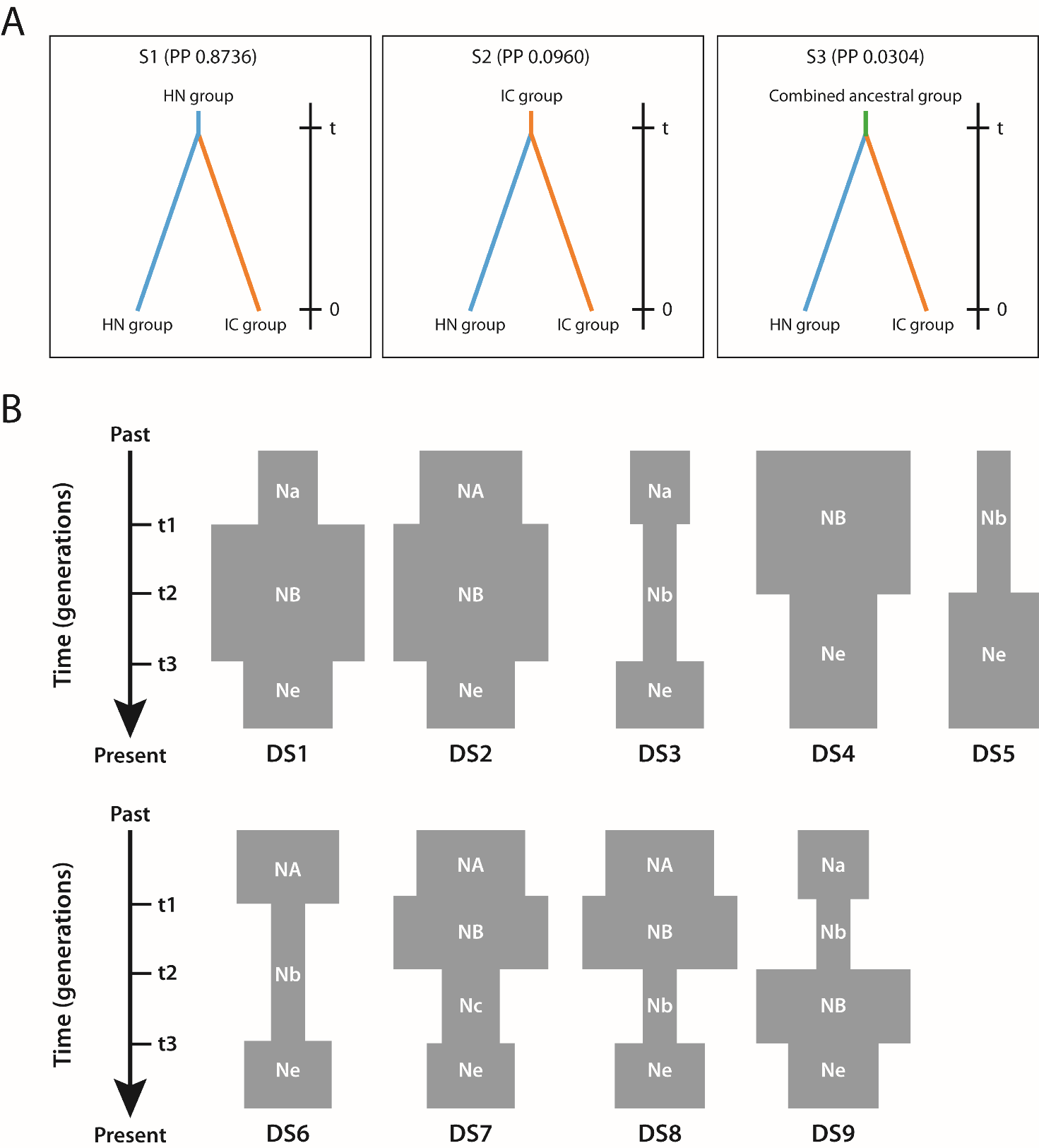


**NA**

**Na**


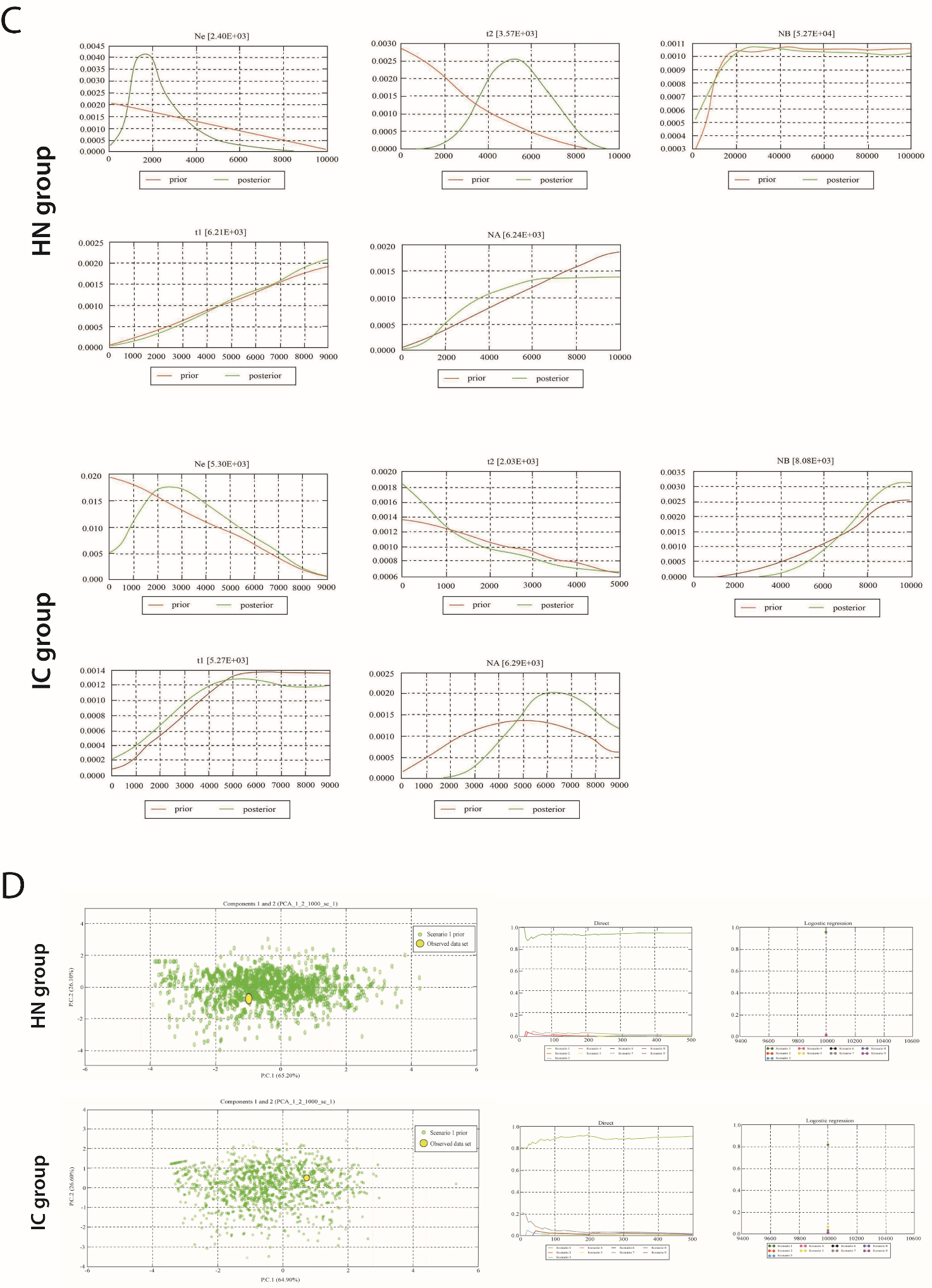


Supplementary File S2. Analysis of the historical demography and ancestral distribution of *Phalaenopsis pulcherrima* using DIYABC. A. The three possible divergence scenarios and their corresponding posterior probabilities: (S1) IC group originated from HN group and diverged at t1, (S2) HN group originated from IC group and diverged at t1, and (S3) both HN and IC groups originated from a single combined HN+IC ancestral population and diverged at t1. B. Nine potential demographic scenarios of population size change: (DS1) expansion + contraction model, Ne < NA, (DS2) expansion + contraction model, Ne > Na, (DS3) contraction + expansion model, Ne > Na, (DS4) old contraction model, Ne < NA, (DS5) old expansion model, Ne > Na, (DS6) contraction + expansion model, Ne < NA, (DS7) old expansion + intermediate contraction + recent expansion, Ne < NA, Ne > Nc > Na, (DS8) old expansion + intermediate contraction + recent expansion, Ne < NA, Ne > Na > Nb, and (DS9) old contraction + intermediate expansion + recent contraction, Ne > Na > Nb. NA indicates large ancestral population size, Na indicates small ancestral population size and Ne indicates current population size, with the relationship between different population sizes being NB > NA > Ne > Nc > Na > Nb; t1 indicates old expansion or contraction time, t2 indicates intermediate expansion or contraction time and t3 indicates recent expansion or contraction time, with t1 > t2 > t3. C. Prior and posterior distribution of demographic parameters based on the best-fit scenarios for the HN group and IC group. D. PCA plots to check fitness of simulated and observed data sets for the two groups based on direct logistic regression of the best-fit scenarios. Unfilled green circles indicate data simulated with parameters drawn from prior distributions, filled green circles indicate data simulated with parameters drawn from posterior distributions, and yellow circles indicate observed data. Scenarios 1–9 correspond to DS1–DS9 shown in part B, respectively.
