# Supplementary material for: Exploring island syndromes: Variable matrix permeability in Phalaenopsis pulcherrima (Orchidaceae), a specialist lithophyte of tropical Asian inselbergs
Source: Front Plant Sci. 2023 Feb 20;14:1097113. doi: 10.3389/fpls.2023.1097113 (PMC9986494; doi:10.3389/fpls.2023.1097113)
Supplement: Supplementary file 3 [file Table_3.docx]

Supplementary File S3. STRUCTURE output implementing the Evanno method (Evanno et al., 2005; Earl and VonHoldt, 2012).

| K | Reps | Mean LnP(K) | Stdev LnP(K) | Ln'(K) | \|Ln''(K)\| | Delta K |
| --- | --- | --- | --- | --- | --- | --- |
| 1 | 20 | -16310.605 | 0.785711 | — | — | — |
| 2 | 20 | -14502.92 | 0.817956 | 1807.685 | 1201.515 | 1468.923083 |
| 3 | 20 | -13896.75 | 157.030052 | 606.17 | 250.685 | 1.596414 |
| 4 | 20 | -13541.265 | 122.975831 | 355.485 | 439.05 | 3.570214 |
| 5 | 20 | -13624.83 | 1116.872274 | -83.565 | 628.695 | 0.562907 |
| 6 | 20 | -13079.7 | 69.301819 | 545.13 | 530.51 | 7.655066 |
| 7 | 20 | -13065.08 | 339.657255 | 14.62 | 20.505 | 0.06037 |
| 8 | 20 | -13029.955 | 492.577152 | 35.125 | 180.275 | 0.365983 |
| 9 | 20 | -12814.555 | 167.928671 | 215.4 | 102.98 | 0.613237 |
| 10 | 20 | -12702.135 | 357.773376 | 112.42 | 6.975 | 0.019496 |
| 11 | 20 | -12596.69 | 143.510949 | 105.445 | 228.175 | 1.589948 |
| 12 | 20 | -12719.42 | 528.698016 | -122.73 | 373.44 | 0.706339 |
| 13 | 20 | -12468.71 | 154.277395 | 250.71 | 564.48 | 3.658864 |
| 14 | 20 | -12782.48 | 648.956899 | -313.77 | 387.835 | 0.597628 |
| 15 | 20 | -12708.415 | 612.512785 | 74.065 | 513.905 | 0.839011 |
| 16 | 20 | -13148.255 | 1325.51793 | -439.84 | 638.105 | 0.481401 |
| 17 | 20 | -12949.99 | 830.85659 | 198.265 | 599.76 | 0.721857 |
| 18 | 20 | -13351.485 | 1325.434895 | -401.495 | 152.49 | 0.115049 |
| 19 | 20 | -13905.47 | 1723.060068 | -553.985 | 29.44 | 0.017086 |
| 20 | 20 | -14430.015 | 1844.747379 | -524.545 | — | — |
