# Supplementary material for: Exploring island syndromes: Variable matrix permeability in Phalaenopsis pulcherrima (Orchidaceae), a specialist lithophyte of tropical Asian inselbergs
Source: Front Plant Sci. 2023 Feb 20;14:1097113. doi: 10.3389/fpls.2023.1097113 (PMC9986494; doi:10.3389/fpls.2023.1097113)
Supplement: Supplementary file 4 [file Table_4.docx]

Supplementary File S4. Bottleneck test for *Phalaenopsis pulcherrima* populations under the TPM model.

| Region | Population | No. of individuals | Probability |
| --- | --- | --- | --- |
| Hainan Island (HN) | WX1 | 56 | 0.670 |
|  | WX2 | 39 | 0.017^*^ |
|  | WX3 | 60 | 0.030^*^ |
|  | YJ | 12 | 0.204 |
|  | DL | 5 | 0.966 |
|  | DLH | 5 | 0.091 |
|  | JF | 6 | 0.204 |
|  | LD1 | 16 | 0.952 |
|  | LD2 | 9 | 0.850 |
| HN group | Total | 208 | 0.049^*^ |
| Mainland Indochina (IC) | YD | 2 | — |
|  | PKK | 13 | 0.005^*^ |
|  | DPV | 2 | — |
|  | NPA1 | 4 | 0.946 |
|  | NPA2 | 40 | 0.463 |
|  | PXH1 | 23 | 0.808 |
|  | PXH2 | 7 | 0.414 |
|  | XP | 4 | 0.048^*^ |
|  | PHR | 9 | 0.008^*^ |
|  | PR | 9 | 0.583 |
|  | KRS | 2 | — |
| IC group | Total | 115 | 0.025^*^ |

Dash (—) indicates that probability information was not available (due to n<3). Significance is indicated as * *p* <0.05.
