# Supplementary material for: Exploring island syndromes: Variable matrix permeability in Phalaenopsis pulcherrima (Orchidaceae), a specialist lithophyte of tropical Asian inselbergs
Source: Front Plant Sci. 2023 Feb 20;14:1097113. doi: 10.3389/fpls.2023.1097113 (PMC9986494; doi:10.3389/fpls.2023.1097113)
Supplement: Supplementary file 5 [file Table_5.docx]

Supplementary File S5. Log (marginal likelihood), log (Bayes factor) and model probability of the six models (M1–M6) as computed in MIGRATE.

| Model | Log (marginal likelihood) | Log (Bayes factor) | Model probability |
| --- | --- | --- | --- |
| M1 | -1595.59 | -114.72 | 0.00 |
| M2 | -1552.42 | -71.55 | 0.00 |
| M3 | -1493.11 | -12.24 | 0.00 |
| M4 | -1480.87 | 0 | 1.00 |
| M5 | -1767.78 | -286.91 | 0.00 |
| M6 | -1779.69 | -298.82 | 0.00 |
