# Supplementary material for: Exploring island syndromes: Variable matrix permeability in Phalaenopsis pulcherrima (Orchidaceae), a specialist lithophyte of tropical Asian inselbergs
Source: Front Plant Sci. 2023 Feb 20;14:1097113. doi: 10.3389/fpls.2023.1097113 (PMC9986494; doi:10.3389/fpls.2023.1097113)
Supplement: Supplementary file 6 [file Table_6.docx]

Supplementary File S6. DIYABC parameter estimation for all best-fitting divergence and demography scenarios.

|  | Divergence model (S1, PP = 0.8736 [95% CI: 0.8623–0.8849]) | | | | |
| --- | --- | --- | --- | --- | --- |
| Parameter | HN group | IC group | t |  |  |
| Mean | 9.34E+03 | 7.66E+04 | 1.43E+04 |  |  |
| Median | 8.97E+03 | 7.92E+04 | 1.31E+04 |  |  |
| Mode | 8.36E+03 | 8.41E+04 | 1.01E+04 |  |  |
| 95% CI | 3.61E+03 | 4.00E+04 | 1.01E+04 |  |  |
|  | 1.76E+04 | 9.80E+04 | 2.62E+04 |  |  |
|  | Demographic history model of HN group (DS1, PP = 0.9540 [95% CI: 0.9460–0.9620]) | | | | |
| Parameter | Ne | t2 | NB | t1 | NA |
| Mean | 2.40E+03 | 3.57E+03 | 5.27E+04 | 6.21E+03 | 6.24E+03 |
| Median | 2.00E+03 | 3.33E+03 | 5.23E+04 | 6.57E+03 | 6.38E+03 |
| Mode | 1.34E+03 | 2.50E+03 | 1.38E+04 | 8.94E+03 | 8.42E+03 |
| 95% CI | 8.50E+02 | 6.44E+02 | 8.68E+03 | 1.75E+03 | 1.84E+03 |
|  | 6.15E+03 | 7.61E+03 | 9.76E+04 | 8.90E+03 | 9.82E+03 |
|  | Demographic history model of IC group (DS1, PP = 0.8126 [95% CI:0.7824–0.8428]) | | | | |
| Parameter | Ne | t2 | NB | t1 | NA |
| Mean | 5.30E+03 | 2.03E+03 | 8.08E+03 | 5.27E+03 | 6.29E+03 |
| Median | 5.26E+03 | 1.83E+03 | 8.36E+03 | 5.35E+03 | 6.36E+03 |
| Mode | 5.14E+03 | 2.36E+01 | 9.11E+03 | 5.43E+03 | 6.39E+03 |
| 95% CI | 2.53E+03 | 5.53E+01 | 4.82E+03 | 1.01E+03 | 3.30E+03 |
|  | 8.15E+03 | 4.80E+03 | 9.92E+03 | 8.81E+03 | 8.78E+03 |
